# Elevated tumor NOS2/COX2 promotes immunosuppressive phenotypes associated with poor survival in ER^–^ breast cancer

**DOI:** 10.1172/jci.insight.193091

**Published:** 2025-07-15

**Authors:** Lisa A. Ridnour, Robert Y.S. Cheng, William F. Heinz, Milind Pore, Ana L. Gonzalez, Elise L. Femino, Rebecca L. Moffat, Adelaide L. Wink, Fatima Imtiaz, Leandro L. Coutinho, Donna Butcher, Elijah F. Edmondson, M. Cristina Rangel, Stephen T.C. Wong, Stanley Lipkowitz, Sharon A. Glynn, Michael P. Vitek, Daniel W. McVicar, Xiaoxian Li, Stephen K. Anderson, Nazareno Paolocci, Stephen M. Hewitt, Stefan Ambs, Timothy R. Billiar, Jenny C. Chang, Stephen J. Lockett, David A. Wink

**Affiliations:** 1 Cancer Innovation Laboratory, Center for Cancer Research, National Cancer Institute, NIH, Frederick, Maryland, USA.; 2 Optical Microscopy and Analysis Laboratory, Cancer Research Technology Program, and; 3 Imaging Mass Cytometry, Frederick National Laboratory for Cancer Research, Frederick, Maryland, USA.; 4 Optical Microscopy and Analysis Laboratory, Office of Science and Technology Resources, Center for Cancer Research, National Cancer Institute, NIH, Frederick, Maryland, USA.; 5 Center for Translational Research in Oncology, ICESP/HC, Faculdade de Medicina da Universidade de São Paulo and Comprehensive Center for Precision Oncology, Universidade de São Paulo, São Paulo, Brazil.; 6 Molecular Histopathology Laboratory, Frederick National Laboratory for Cancer Research, National Cancer Institute, Frederick, Maryland, USA.; 7 Houston Methodist Neal Cancer Center, Weill Cornell Medical College, Houston Methodist Hospital, Houston, Texas, USA.; 8 Women’s Malignancies Branch, Center for Cancer Research, National Cancer Institute, NIH, Bethesda, Maryland, USA.; 9 Discipline of Pathology, Lambe Institute for Translational Research, School of Medicine, University of Galway, Galway, Ireland.; 10 Department of Neurology, School of Medicine, Duke University, Durham, North Carolina, USA.; 11 Department of Pathology and Laboratory Medicine, Emory University, Atlanta, Georgia, USA.; 12 Basic Science Program, Frederick National Laboratory for Cancer Research, Frederick, Maryland, USA.; 13 Division of Cardiology, Department of Medicine, Johns Hopkins University, Baltimore, Maryland, USA.; 14 Dipartimento di Scienze Biomediche, Universita di Padova, Padua, Italy.; 15 Laboratory of Pathology and; 16 Laboratory of Human Carcinogenesis, Center for Cancer Research, National Cancer Institute, NIH, Bethesda, Maryland, USA.; 17 Department of Surgery, University of Pittsburgh Medical Center, Pittsburgh, Pennsylvania, USA.

**Keywords:** Inflammation, Oncology, Adaptive immunity, T cells

## Abstract

Tumor immunosuppression affects survival and treatment efficacy. Tumor NOS2/COX2 coexpression strongly predicts poor outcome in estrogen receptor–negative (ER^–^) breast cancer by promoting metastasis, drug resistance, cancer stemness, and immune suppression. Herein, a spatially distinct NOS2/COX2 and CD3^+^CD8^+^PD1^–^ T effector (T_Eff_) cell landscape correlated with poor survival in ER^–^ tumors. NOS2 was primarily expressed at the tumor margin, whereas COX2 together with B7H4 was associated with immune desert regions lacking T_Eff_ cells, where a higher ratio of tumor NOS2 or COX2 to T_Eff_ cells predicted poor survival. Also, programmed cell death ligand 1/programmed cell death 1, regulatory T cells (T_Regs_), and IDO1 were primarily associated with stroma-restricted T_Eff_ cells. Regardless of the survival outcome, CD4^+^ T cells and macrophages were primarily in stromal lymphoid aggregates. Finally, in a 4T1 model, COX2 inhibition led to increased CD8^+^ T_Eff_/CD4^+^ T_Reg_ ratio and CD8^+^ T_Eff_ infiltration while Nos2 deficiency had no significant effect, thus reinforcing our observations that COX2 is an essential component of immunosuppression through CD8^+^ T_Eff_ cell exclusion from the tumor. Our study indicates that tumor NOS2/COX2 expression plays a central role in tumor immune evasion, suggesting that strategies combining clinically available NOS2/COX2 inhibitors with immune therapy could provide effective options for the treatment of aggressive and drug-resistant ER^–^ breast tumors.

## Abstract 2

Elevated tumor NOS2/COX2 expression play a central role in tumor immune evasion, which can be targeted using clinically available inhibitors for improved clinical outcomes.

## Graphical abstract

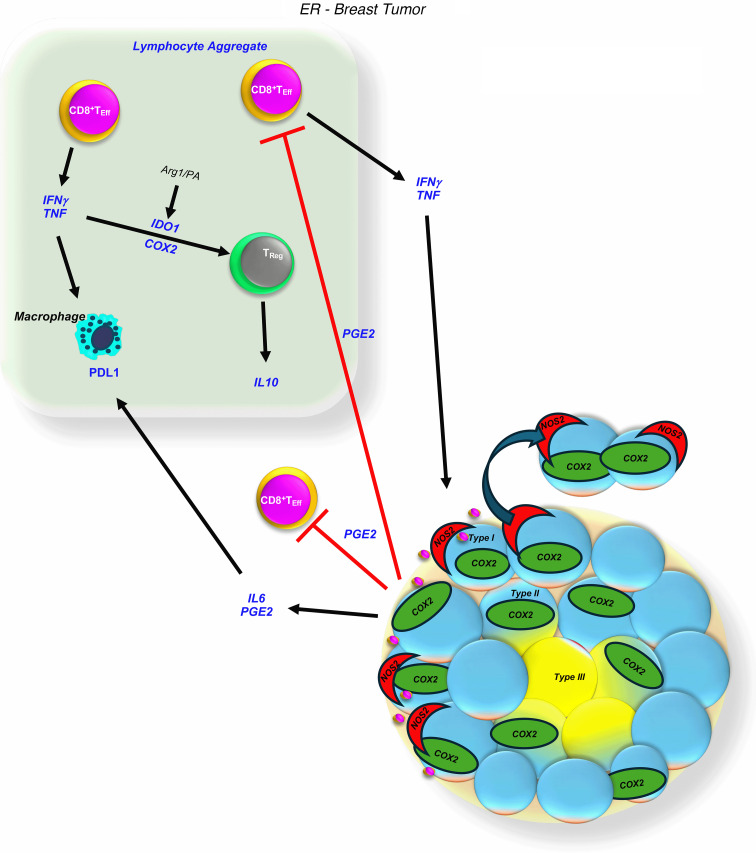

## Introduction

Immune therapies, such as checkpoint inhibitors, cancer vaccines, and T cell adoptive therapies, have created novel treatment options for advanced cancers (1–6). Immune and conventional combination therapies have shown that correct immune polarization is a key factor in the effective treatment of aggressive tumors. Various immunosuppressive mechanisms within the tumor microenvironment (TME) provide targetable stumbling blocks for successfully treating challenging cancers (7). Given the importance of immune evasion for tumor survival and disease progression, the spatial characterization of immune phenotypes could identify novel therapeutic targets. From a spatial perspective, numerous molecular effectors inhibiting correct immune polarization and antitumor immune response within the TME act on the immune, stromal, and tumor cell compartments. Pro-tumor immunosuppressive mechanisms include T cell–centered mechanisms, such as programmed cell death 1/programmed cell death ligand 1 (PD1/PDL1) and regulatory T cells (T_Regs_), cellular factors, cytokines, growth factors, and small molecules derived from metabolites, such as kynurenines and polyamines (8–11). The coexistence of these different immunosuppressive pathways in the TME provides multiple layers of immune suppression.

NOS2 (inducible NOS) and COX2 promote cancer disease progression and poor survival (12–17) and play roles during immune suppression in several cancers. In many solid tumors, NOS2 and COX2 are elevated (18–23). In estrogen receptor–negative (ER^–^) breast cancer, in particular, elevated NOS2/COX2 levels are strongly predictive of poor prognosis (16). Both NOS2 and COX2 form a feed-forward loop fueling the propagation of different pro-tumorigenic mechanisms. They, in turn, activate primary oncogenic pathways, ultimately leading to metastasis, chemoresistance, and cancer stemness (16). According to recent research (12, 24), NOS2 and COX2 are associated with the stroma restriction of CD8^+^ T cells in human malignancies. Moreover, NOS2/COX2 inhibition produced profound changes in antitumor immunity that reduced both metastatic burden and tumor growth and led to increased cures and resistance to tumor rechallenge in the aggressive 4T1 mouse model (24). Importantly, NOS/COX inhibitors provide potential options for improved survival in chemoresistant triple-negative breast cancer (TNBC) (24–27).

Mechanistically, NOS2 and COX2 induce IL-10, TGF-β, and IL-6, which are immunosuppressive within the TME (24, 28, 29) and modify cellular metabolic programming (16, 30–32). NOS2 and COX2 are heme proteins that can swiftly respond to environmental changes, such as hypoxia and inflammation, making them crucial components linking environmental sensing and immune status (33). Stroma-restricted CD8^+^ T cells and IFN-γ are essential for tumor NOS2/COX2 expression (12). This requirement of antitumor factors to induce NOS2/COX2 is strongly associated with poor survival, which presents a conundrum. Indeed, elevated NOS2 and COX2 at tumor margins are close to stroma-restricted CD8^+^ T cells and lymphoid aggregates, whereas, in cold regions (i.e., low CD8^+^ T cell counts) and immune deserts, COX2 is present (24). However, when CD8^+^ T cells penetrate the tumor nest, NOS2/COX2 levels in the entire tumor are low, suggesting their role(s) in CD8^+^ T cell restriction and exclusion from the tumor core. This progression from an inflammatory region to immune desert status suggests that NOS2 and COX2 could provide therapeutic targets that limit the formation of tumor immune deserts.

IFN-γ and cytokines are essential for tumor eradication, while IFN-γ–induced NOS2/COX2 induction is linked to poor prognoses (12, 25). This apparent dichotomy of IFN-γ activity between tumor eradication (facilitated by host antitumor immune responses) and NOS2/COX2-associated immunosuppression implicates it as a key modulator in a delicate balance between tumor immune surveillance and immune evasion (16, 34, 35). Similar dichotomies exist for the immunosuppressive agents IDO1 and PDL1 that are stimulated by IFN-γ (36, 37). This negative feedback response to IFN-γ results in suppression of cytolytic T cell activity. Thus, the spatial evaluation of immunosuppressive factors can be exploited for improved clinical outcomes.

In the current study, we performed IHC analyses on ER^–^ breast cancer biopsies to determine whether the spatial distribution of tumor NOS2 and COX2 contributes to immune-suppressive mechanisms involving T_Regs_, PD1/PDL1, IDO1, and B7H4 to suppress T effector (T_Eff_) cell function. The evidence herein demonstrates that the spatial orientation of immune and tumor cells in conjunction with tumor NOS2/COX2 creates a unique tumor landscape that drives the formation of immune deserts and immune cell restriction, fueling immune suppression, tumor immune evasion, and cancer disease progression.

## Results

### Immunosuppressive markers and patient survival.

**Figure 1:**
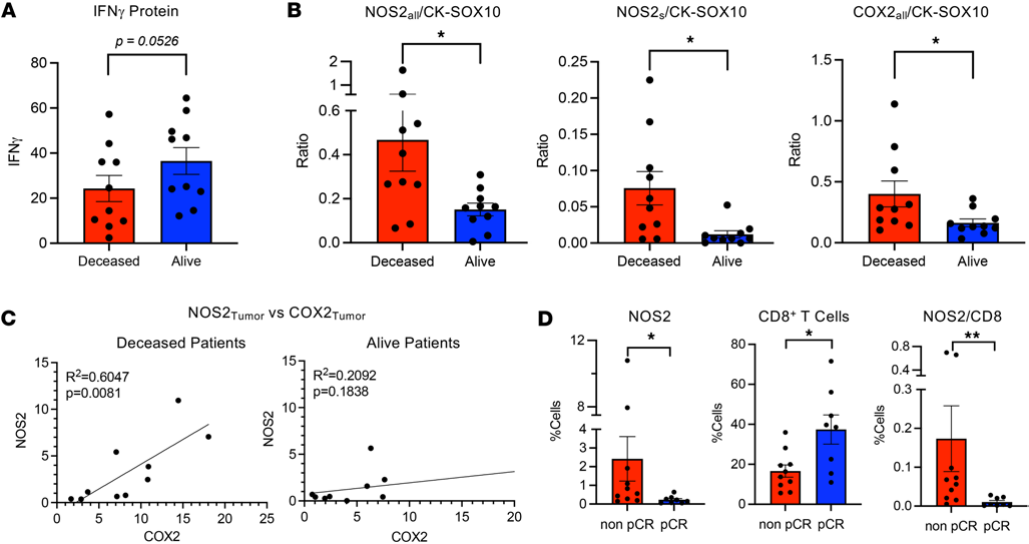
Survival analysis of tumor NOS2, COX2, and IFN-γ at 5 years after diagnosis. Annotated viable tumor was analyzed for survival effects of (**A**) IFN-γ and (**B**) tumor NOS2 and COX2 normalized to CK-SOX10 tumor biomarker in tumors from deceased (*n* = 10) and alive (*n* = 10) ER^–^ breast cancer patients at 5 years after diagnosis. (**C**) Pearson’s correlation analysis of NOS2 and COX2 in tumors from deceased and alive ER^–^ breast cancer patients. (**D**) Effect of tumor NOS2 and CD8^+^ T cells on complete pathological response (non pCR *n* = 10, pCR *n* = 8) in biopsies from patients in the neoadjuvant KEYNOTE 522 (K522) clinical trial who received the PD1 inhibitor pembrolizumab and chemotherapy. *P < 0.05, **P = 0.0031, Mann-Whitney test.

Herein, immunosuppressive mechanisms of tumor NOS2/COX2 expression were spatially explored using multiplex immunofluorescence imaging of 20 ER^–^ tumors from patients who succumbed to disease (deceased; *n* = 10) versus those who survived (alive; *n* = 10) at 5 years after diagnosis. Patient data are summarized in Table 1. Multiple immune cell markers were evaluated, including T cell markers, CD3, CD4, CD8; the macrophage marker CD68; CK-SOX10 tumor marker; and immune modulatory factors, PD1, PDL1, FOXP3, IFN-γ, NOS2, and COX2. Univariate analysis of the percentage of cells containing these markers was compared with 5-year survival status where IFN-γ trended higher in surviving patients (Figure 1A). Prior studies have shown that NOS2 is predominantly associated with the tumor epithelium (38). Annotated regions of viable tumor were analyzed. When normalized to the CK-SOX10 tumor biomarker, an elevated tumor NOS2 expression gradient (12) defined by weak, medium, or strong (NOS2s) expression levels was observed that correlated with poor outcome (Figure 1B). Tumor COX2 expression normalized to CK-SOX10 was also elevated in tumors from deceased patients (Figure 1B). These results were supported by a strong correlation between tumor-associated NOS2 and COX2 expression in deceased patient tumors only (Figure 1C). The expression levels of other biomarkers examined did not change significantly with respect to survival (data not shown). These results suggest that elevated tumor NOS2 and COX2 expression promotes disease progression and poor ER^–^ breast cancer survival. Thus, elevated tumor NOS2/COX2 at the single-cell level is predictive of poor clinical outcome and supports previous IHC-based studies (38, 39). The importance of these NOS2, COX2, and CD8 relationships was further explored with respect to clinical trial outcomes in biopsies from patients in the neoadjuvant KEYNOTE 522 clinical trial who received the PD1 inhibitor pembrolizumab and chemotherapy. Elevated tumor NOS2 expression strongly correlated with poor outcome (non pCR *n* = 10) as defined by pathological complete response (pCR *n* = 8) (Figure 1D), while increased CD8^+^ T cells were predictive of pCR (Figure 1D). COX2 had no predictive value in this cohort. The ratio of NOS2/CD8 demonstrated a strong predictive relationship (Figure 1D) and was consistent with Figure 1B. This result demonstrates the predictive power of the NOS2/CD8 relationship with respect to survival and treatment efficacy (27).

**Figure 2:**
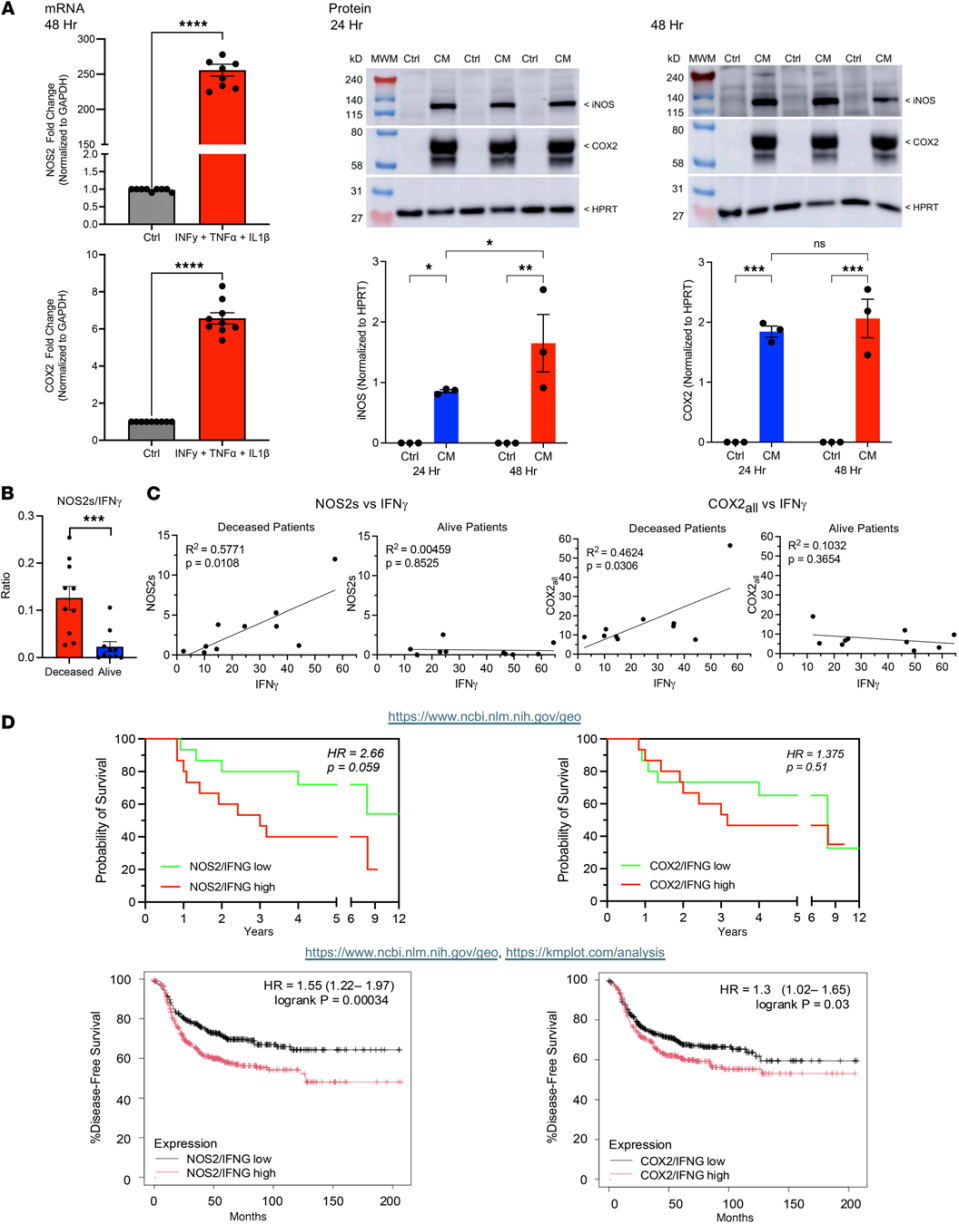
Cytokine-induced NOS2 and COX2 expression. (**A**) Gene expression (left) and Western blot (right) showing temporal NOS2 and COX2 expression in MDA-MB-231 breast cancer cells treated with IFN-γ + IL-1β + TNF-α (CM) at 24 and 48 hours. ****P < 0.0001 Mann-Whitney test for both NOS2 (top) and COX2 (bottom); 2-way ANOVA + Fisher’s least significant difference *P < 0.05, **P = 0.0012, or Šídák's multiple-comparison test ***P = 0.0006. MWM, molecular weight marker. (**B**) NOS2s/IFN-γ ratio is significantly elevated in tumors from deceased ER^–^ patients at 5 years after diagnosis. ***P = 0.0007 Mann-Whitney test. (**C**) Pearson’s correlation showing significance between NOS2s and COX2_all_ versus IFN-γ in tumors from deceased and alive patients at 5 years after diagnosis. (**D**) The significance of the NOS2/IFNG and COX2/IFNG relationships are validated in National Center for Biotechnology Information (NCBI) Gene Expression Omnibus (GEO) datasets found in https://www.ncbi.nlm.nih.gov/geo/ Gehan-Breslow-Wilcoxon survival analysis and Mantel-Haenszel hazard ratio analysis were used. Top graphs validate survival using cohort data from this study, while lower graphs are from combined GEO datasets (*n* = 796) generated using https://kmplot.com/analysis

We have recently demonstrated a requirement of IFN-γ–associated with stroma-restricted CD8^+^ T cells for tumor NOS2 and COX2 expression in ER^–^ breast tumors (12) that is higher in tumors from deceased patients (40). Moreover, IFN-γ, IL-1β, and TNF-α are key cytokines released by cytotoxic CD8^+^ T_Eff_ cells (41); in vitro exposure to IFN-γ, IL-1β, and TNF-α induced cellular elongation, migration, and invasion of ER^–^ MDA-MB-231 breast cancer cells, which were limited by NOS/COX inhibitors (12). Using these conditions, the temporal expression of NOS2 and COX2 induction in MDA-MB-231 cells was explored by gene expression and Western blotting. Figure 2A shows elevated NOS2/COX2 message at 48 hours and protein at 24 and 48 hours in IFN-γ + IL-1β + TNF-α–treated MDA-MB-231 cells. These results are consistent with elevated tumor NOS2 and COX2 in the tumor immune microenvironment and is consistent with the NOS2/COX2 feed-forward loop (25). In support of these findings, we observed a significantly increased NOS2s/IFN-γ ratio in tumors from deceased patients (Figure 2B). In addition, significant correlations between NOS2s and IFN-γ, as well as COX2 and IFN-γ, were observed in tumors from deceased patients while no significance was observed in tumors from surviving (alive) patients at 5 years after diagnosis (Figure 2C). While COX2 is important for immunosuppressive landscapes, the importance of the NOS2/IFN-γ relationship pertains to the spatial localization of IFN-γ secreted from stroma-restricted CD8^+^ T cells (12), which then induces NOS2 tumor expression at the tumor margin (40). NO promotes tumor cell migration and invasion and has been localized to metastatic niches (38, 40, 42). NOS2-derived NO induces COX2/prostaglandin E_2_ (PGE2), which then promotes NOS2 feed-forward signaling (25). Therefore, we validated both the NOS2/IFNG and COX2/IFNG relationships in our cohort obtained from the NCBI GEO database (https://www.ncbi.nlm.nih.gov/geo/) [RRID:SCR_005012] and compiled GEO transcriptomic ER^–^ datasets (*n* = 796) as reported by B Gyorffy (43). These analyses demonstrate the predictive power of NOS2/IFNG and COX2/IFNG relationships in ER^–^ breast cancer (Figure 2D).

We have recently observed that elevated tumor NOS2 and COX2 limits pro-inflammatory CD8^+^ T cell infiltration into tumors from deceased patients (40), which could promote an immunosuppressive TME. To further explore survival relationships between tumor NOS2 and COX2 expression relative to T cells, PD1, PDL1, and FOXP3 immunosuppressive markers, we evaluated the ratios of the percentage of cells expressing each marker to tumor NOS2 or COX2. Interestingly, when we normalized to tumor NOS2 or COX2 expression (NOS2_Tumor_, COX2_Tumor_), we found significant increases in both exhausted and regulatory CD8^+^ T cells (CD8^+^T_Ex_ and CD8^+^T_Reg_, respectively), as well as PDL1 and PDL1_Tumor_/NOS2_Tumor_ and CD4^+^T_Reg_/NOS2_Tumor_ ratios in tumors from alive patients (Supplemental Figure 1, A–C; supplemental material available online with this article; https://doi.org/10.1172/jci.insight.193091DS1). These results suggest relationships between tumor NOS2/COX2 and both exhausted and regulatory T cell phenotypes in ER^–^ breast cancer.

### NOS2 and COX2 are predominant in immune cold tumor regions.

**Figure 3:**
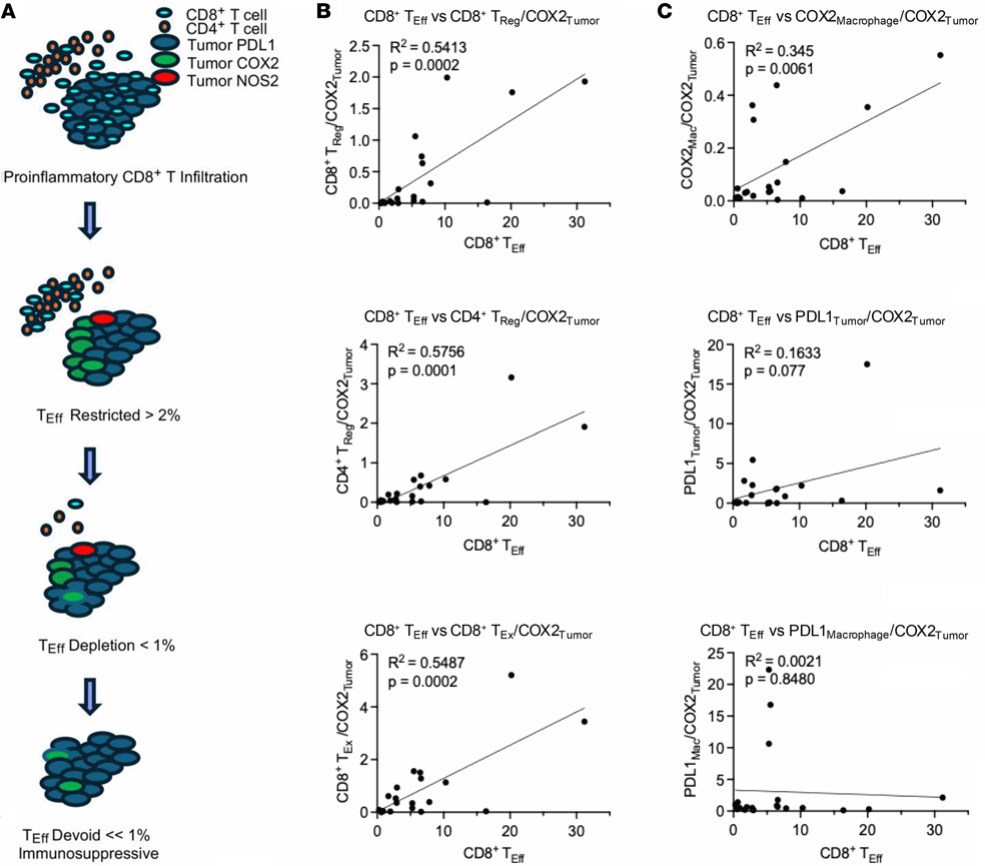
Tumor COX2 promotes immune cold regions. (**A**) Schematic showing immunosuppressive mediators and abated CD8^+^ T_Eff_ cell infiltration. Pearson’s correlation analysis showing significance between CD8^+^ T_Eff_ cells versus immunosuppressive phenotypes normalized to COX2 tumor expression, where (**B**) CD8^+^ T_Reg_, CD4^+^ T_Reg_, and CD8^+^ T_Ex_ cells, as well as (**C**) COX2-expressing macrophages but not PDL1-expressing tumor macrophages correlated significantly.

The predictive value of spatially distinct CD8^+^ T cells has been reported (24, 40, 44). Cytotoxic T_Eff_ cells are known to secrete high levels of IFN-γ as part of a pro-inflammatory antitumor response. Abated CD8^+^ T cell tumor infiltration is associated with elevated tumor COX2 expression and an immunosuppressive signature (24) as summarized in Figure 3A. Our analyses show that COX2 is predominantly (93.5%) expressed by the tumor. Given that elevated tumor COX2 inhibited CD8^+^ T cell tumor infiltration (24, 40), correlations with other immunosuppressive mediators including CD4^+^ and CD8^+^ T_Reg_, CD8^+^ T_Ex_, as well as tumor- and CD68^+^ macrophage–associated PDL1 (PDL1_Tumor_ and PDL1_Macrophage_, respectively) phenotypes were explored using Pearson’s correlation analysis. To demonstrate the significance of the relationships between these immunosuppressive phenotypes, we normalized their expression to tumor COX2 and plotted against CD8^+^ T_Eff_ (CD3^+^CD8^+^PD1^–^) cells. This analysis demonstrated significant correlations for CD8^+^ T_Reg_, CD4^+^ T_Reg_, and exhausted CD8^+^ T_Ex_ cells (Figure 3B). In addition, a positive correlation between COX2_Macrophage_ and CD8^+^ T_Eff_ phenotypes was observed. However, no such correlation was observed between PDL1_Tumor_ or PDL1_Macrophage_ phenotypes and CD8^+^ T_Eff_ cells (Figure 3C). Thus, leukocyte-based immunosuppressive cells are favored in the presence of elevated T_Eff_ cell infiltration in inflamed tumors, while NOS2 and COX2 predominate in immune cold or immune desert regions, indicating distinct roles during tumor immunosuppression.

### Spatial clustering of NOS2^+^ and COX2^+^ tumor phenotypes determines survival in ER^–^ breast cancer.

**Figure 4:**
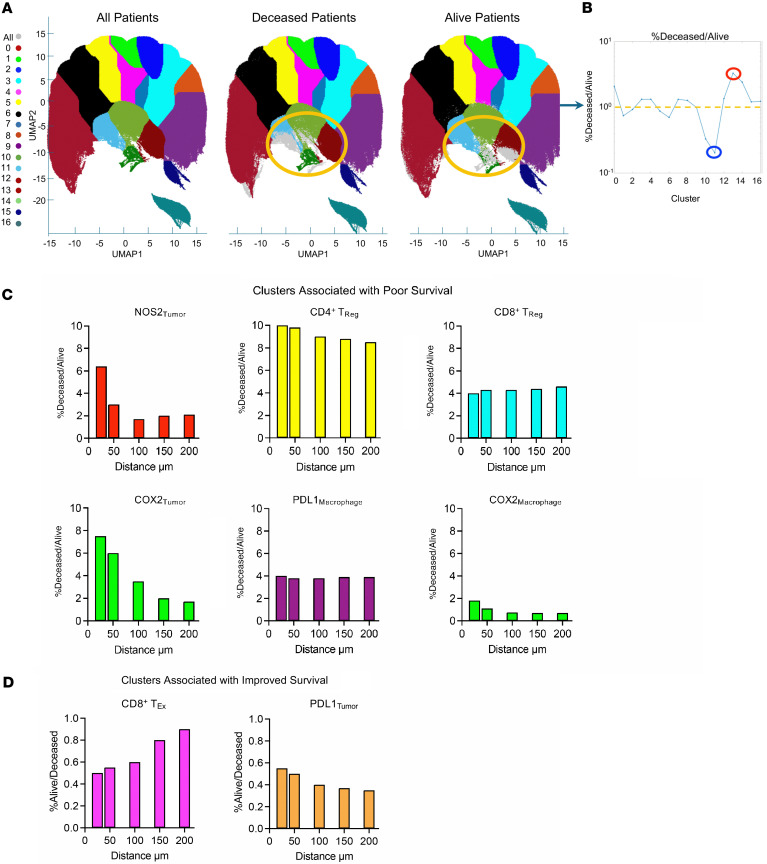
Unsupervised spatial S-UMAP analysis validates the predictive value of NOS2^+^/COX2^+^ tumor clusters. (**A**) S-UMAP of the clustering cellular phenotypes revealing distinct clustering regions (yellow circles) and (**B**) the differential cluster distribution analysis of the of %deceased normalized to %alive (red and blue circles) patient tumors at 5 years after diagnosis. (**C**) Nearest neighborhood analyses show distance-dependent changes in cellular phenotypes that are predictive of poor patient survival. Importantly, density-dependent clustering gradients are demonstrated for NOS2_Tumor_ and COX2_Tumor_ phenotypes. (**D**) Moderately increased clustering of CD8^+^ T_Ex_ and PDL1_Macrophage_ phenotypes is predictive of improved patient survival. For C and D the first bar is 25 µm.

Next, a spatial uniform manifold approximation and projection (S-UMAP) analysis of single-cell neighborhood clusters was conducted (45) to explore the predictive power of immunosuppressive phenotypes, which revealed distinct clustering in the S-UMAP from tumors of deceased versus alive patients at 5 years of survival (Figure 4A, yellow circles). A differential cluster distribution of the ratio of clustered populations by survival status (Figure 4B, red and blue circles) revealed that deceased patient tumors had a greater percentage of *distance-dependent* NOS2^+^ and COX2^+^ tumor clusters (Figure 4C). In addition, CD4^+^ and CD8^+^ T_Reg_ and CD68^+^PDL1^+^ macrophage (PDL1_Macrophage_) phenotypes were elevated in tumors from deceased patients (Figure 4C). Evaluation of the influence of distance between individual phenotypes revealed tumor NOS2^+^ and COX2^+^ gradients (Figure 4C), where the correlation with immunosuppressive cell types decreased rapidly with distance over 100 μm in deceased patient tumors. Importantly, the distance-dependent gradient implicates the predictive power of spatially distinct tumor NOS2/COX2 clustering. In contrast, distance-dependent gradients were not observed in T_Reg_ or PDL1_Macrophage_ and COX2_Macrophage_ phenotypes (Figure 4C). Furthermore, CD8^+^PD1^+^ T_Ex_ and PDL1_Tumor_ phenotypes were marginally elevated and predictive of improved survival (Figure 4D), which could be attributed to increased CD8^+^ T cell tumor infiltration and elevated IFN-γ production that is known to induce PD1 expression (44, 46). This unbiased analysis spatially identifies targetable biomarkers that promote immunosuppressive cellular neighborhoods with predictive value, which include increases in both NOS2^+^ and COX2^+^ tumor clusters in spatially distinct cellular regions that drive immunosuppression and ER^–^ breast tumor metastasis.

### Immune-suppressive mechanisms occur in specific regions of the TME.

**Figure 5:**
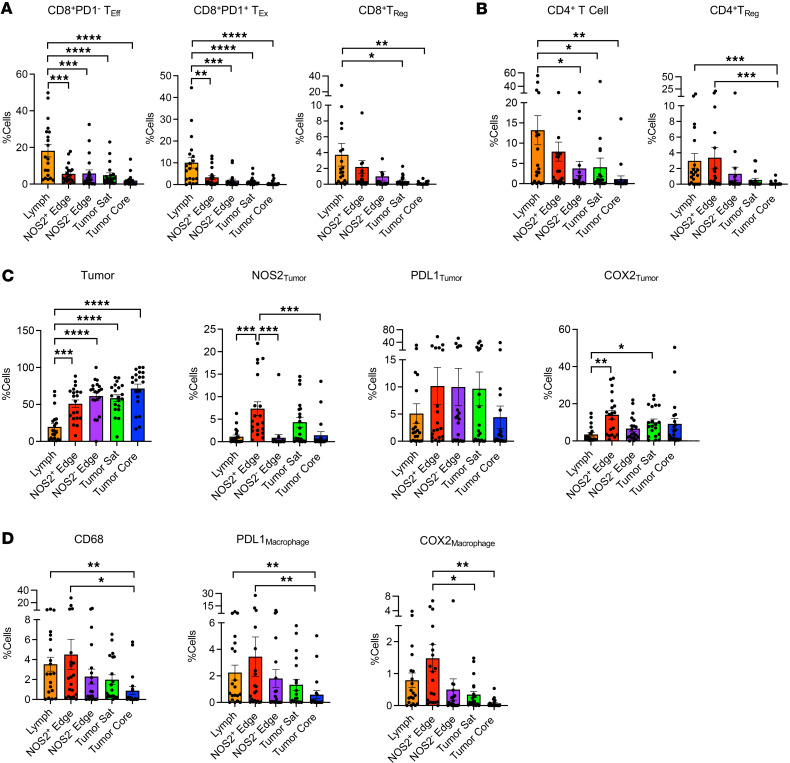
Regional placement of cellular phenotypes within the tumor. The %cells of predictive phenotypes including (**A**) CD8^+^ and (**B**) CD4^+^ (**C**) tumor and (**D**) macrophage phenotypes are shown. Ordinary 1-way ANOVA *P < 0.05, **P ≤ 0.0061, ***P ≤ 0.0004, ****P < 0.0001. Sat, satellites.

Previous reports have demonstrated the predictive value of the spatial architecture with respect to tumor-excluded (cold) versus -infiltrating (hot) CD8^+^ T cells (24, 44, 46). The tumor has numerous spatially distinct regions that could affect tumor-immune interactions (40), which include lymphoid aggregates and small and large tumor nests. Next, the relationship between T_Eff_ and immunosuppressive phenotypes shown in Figure 3 was spatially explored in all tumors. Tumor regions were subdivided by tumor size and tumor NOS2^+/–^ edges at the tumor/stromal interface and tumor core (40). Smaller tumor fragments were defined by tumor clusters > 0.02 mm^2^ and in most cases fewer than 3 cells thick in one dimension (40). In contrast, larger tumor nests were defined as > 0.1 mm^2^ with designated tumor core and edges (40). The percentage of immune-suppressive phenotypes was spatially analyzed in each region (Figure 5). The distribution of lymphoid populations formed a gradient ranging from stroma-restricted lymphoid aggregates composed of both CD3^+^CD4^+^ and CD3^+^CD8^+^ T cells to single CD3^+^CD8^+^ T cells that had penetrated NOS2^+/–^ edges and into the tumor satellite and core regions (Figure 5, A and B). In addition, CD8^+^ T_Regs_ were elevated in stromal lymph aggregates (Figure 5A), while CD4^+^ T_Regs_ were also elevated at NOS2^+^ tumor edges when compared with tumor satellite and/or core regions (Figure 5B). The tumor core had considerably lower leukocyte phenotypes (<3%) (Figure 5, A and B). The distribution of tumor cells was mainly associated with NOS2^+/–^ edges as well as tumor satellite and tumor core regions (Figure 5C). Tumor NOS2 expression was generally localized at NOS2^+^ edges and tumor satellite regions (Figure 5C). The spatial distribution of PDL1_Tumor_ expression did not change significantly across analyzed regions, while COX2 expression was primarily localized near NOS2^+^ edges and tumor satellite regions (Figure 5C). Tumors also contained numerous intermingling CD68^+^ and PDL1^+^ macrophages that were significantly elevated in lymphoid aggregates and the NOS2^+^ tumor edge, while COX2^+^ macrophages were associated with NOS2^+^ tumor edges when compared with the tumor core and/or tumor satellite regions (Figure 5D).

### NOS2/COX2 drives T_Eff_ tumor exclusion.

**Figure 6:**
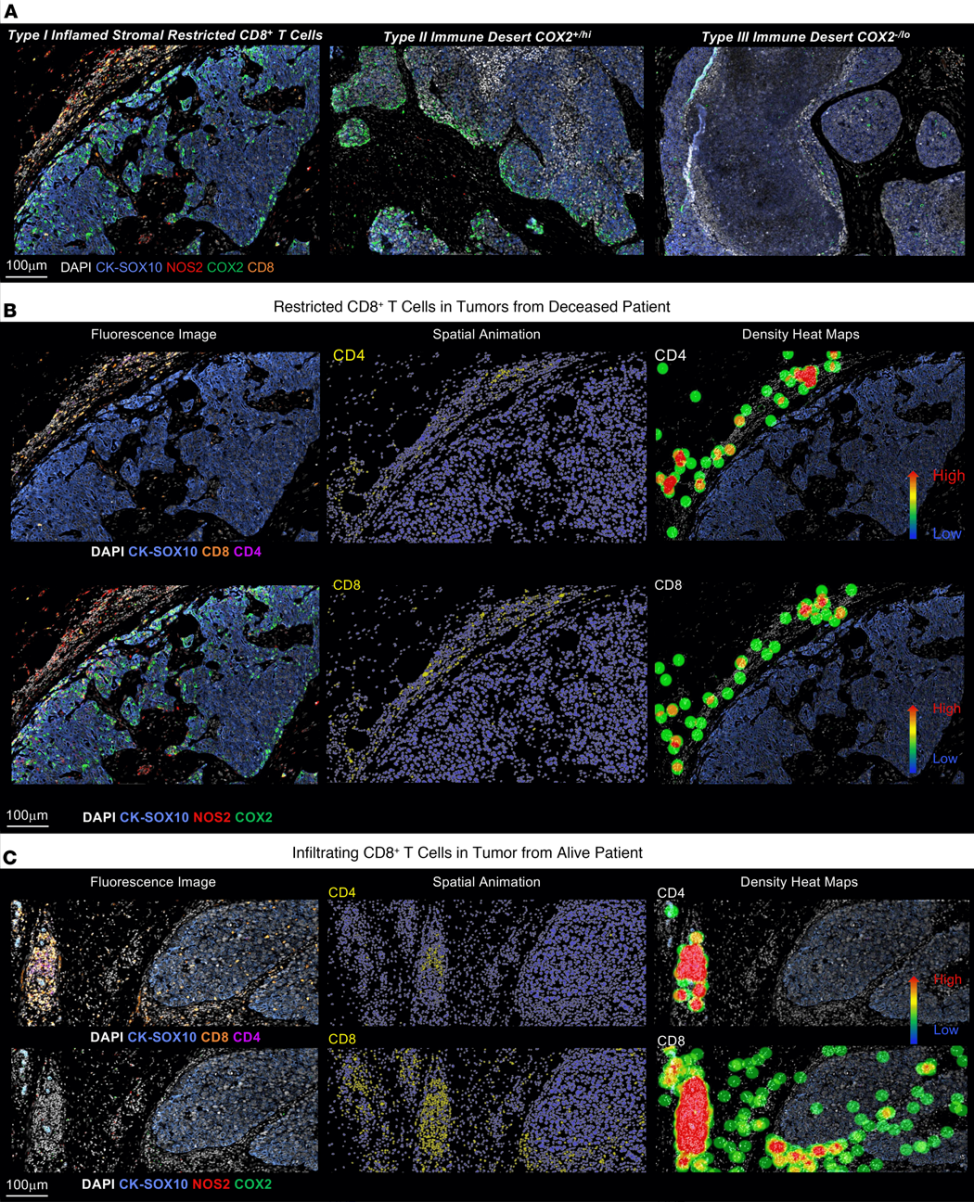
Progression of CD8^+^ T cell exclusion during the formation of tumor immune deserts. Distinct classes of CD8^+^ T cell exclusion were observed. (**A**) Type I: inflamed but stroma-restricted CD8^+^ T cells (orange) with NOS2 (red) expression at the tumor margin and high COX2 (green) expression deeper into tumor core. Type II: abated CD8^+^ T cells, low NOS2 expression at the tumor margin, and high COX2 expression deeper into tumor core. Type III: absence of CD8^+^ T cells with abated NOS2 expression at the tumor margin and low, sparse COX2 expression deeper into tumor core. (**B**) Differential penetration of CD4^+^ versus CD8^+^ T cells into high NOS2/COX2-expressing tumors of deceased patients where both CD4^+^ and CD8^+^ T cells remain in the tumor stroma, as shown in fluorescence image, spatial animation (middle), and density heatmap (right). (**C**) In contrast, CD4^+^ T cells remain in the tumor stroma while CD8^+^ T cells penetrate deep into low NOS2/COX2-expressing tumor core of alive patients.

As previously shown, elevated tumor COX2 limited CD8^+^ T cell infiltration into the tumor, resulting in the development of immune deserts, which involved reduced cytokine and chemokine expression that promotes directional immune cell migration (40). Herein, we extend these observations by comparing different tumor regions, where 3 classes of CD8^+^ T cell exclusion from the tumor nest can be observed during formation of the immune desert: type I restricted inflamed with NOS2^+^ tumor edge and high tumor COX2 expression, type II NOS2^–^ tumor edge with high tumor COX2 expression, and type III with NOS2^–^ tumor edge, low sporadic COX2 expression, and few proximal stromal lymphoid cells < 500 μm (<1%) (Figure 6, A and B). These distinct phenotypes suggest that tumor NOS2/COX2 distributions could have a role in the progression and maintenance of the tumor’s immune deserts and T_Eff_ cell exclusion. Additional biomarkers including CD31, α–smooth muscle actin (α-SMA), collagen IV (stroma), CD20 (B cell), CD3 (T cell), CD14, CD68 (myeloid), and carbonic anhydrase IX (CAH9) (hypoxia) are shown in these regions (Supplemental Figure 2). Interestingly, NOS2 expression is observed in lymphoid aggregates with CD3^+^ T cells in type I immune deserts as well as CD31^+^α-SMA^+^ vessels in type I and type II immune deserts. In contrast, high CD14-expressing myeloid cells were observed in necrotic regions of type III immune deserts. The hypoxia marker CAH9 was also observed in type II immune desert regions.

Our previous work using the murine TNBC 4T1 model demonstrated that CD8^+^ T cells were restricted in the tumor stroma or margins of untreated control mice, similar to type I immune deserts observed in tumors from deceased patients (Figure 6A) (24, 46). While there was less difference between the control and Nos2^–^ animals, inhibition of COX2 using the NSAID indomethacin resulted in extensive tumor infiltration of CD8^+^ T_Eff_ cells and a dramatic increase in IFN-γ (24). Indomethacin treatment also increased CD4^+^ T cells; however, these cells did not infiltrate the tumor but remained at the tumor margin (24, 46). This evidence suggests that while NOS2 and COX2 control the migration of CD8^+^ T cells, CD4^+^ T cell mobility is unaffected but could augment T_Eff_ function at the tumor margin (24, 46). The differential distribution of CD8^+^ and CD4^+^ T cells was also examined in deceased versus alive patient tumors. The tumor edge proximal to lymphoid aggregates was examined in inflamed tumors with stroma-restricted T_Eff_ cells. Limited T cell infiltration was observed in tumors from deceased patients with elevated tumor NOS2/COX2 (Figure 6, A and B). In contrast, low NOS2/COX2-expressing tumors from alive patients exhibited elevated CD8^+^ T cells with abundant infiltration from the tumor-stroma margin deep into the tumor core (Figure 6C). Interestingly, increased CD4^+^ T cell density was observed, which remained in the lymphoid aggregate. These results suggest that COX2 controls CD8^+^ T cell infiltration while CD4^+^ T cells remain in or near the lymphoid aggregate at the tumor margin (Figure 6C).

**Figure 7:**
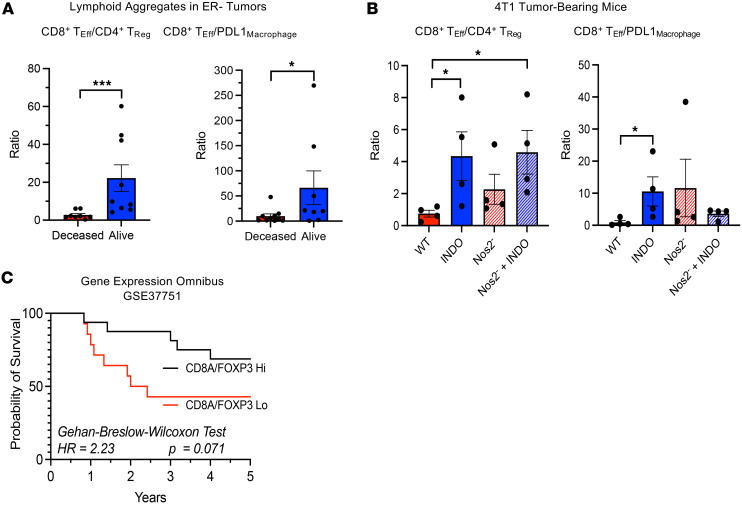
Analysis of immune phenotypes in lymphoid aggregates of ER^–^ tumors. (**A**) CD8^+^ T_Eff_/CD4^+^ T_Eff_ and CD8^+^ T_Eff_/PDL1_Macrophage_ ratios are significantly higher in tumors from alive patients. Statistical outliers were excluded based upon mathematical analysis using Prism software ROUT method. (**B**) CD8^+^ T_Eff_/CD4^+^ T_Reg_ and CD8^+^ T_Eff_/PDL1_Macrophage_ ratios are significantly higher in indomethacin-treated 4T1 tumors when compared with WT control. (**C**) Validation of the predictive value of elevated CD8a/FOXP3 using the GEO database. *P < 0.05, ***P = 0.001 Mann-Whitney test. Survival analysis was performed using Gehan-Breslow-Wilcoxon test, and hazard ratio was determined using Mantel-Haenszel test.

COX2/PGE2 have been shown to favor immune-suppressive T cell phenotypes. Analysis of immune phenotypes in the lymphoid aggregate revealed differences between deceased versus alive patient tumors at 5 years of survival. The impact of T_Eff_ ratio with different immune markers on survival showed elevated CD8^+^ T_Eff_/CD4^+^ T_Reg_ and CD8^+^ T_Eff_/PDL1_Macrophage_ ratios in tumors from surviving patients (Figure 7A). This finding was corroborated in the 4T1 model, where these ratios were significantly increased following indomethacin treatment of WT and Nos2^–^ tumor-bearing mice (Figure 7B), which showed that COX2 inhibition but not NOS2 deficiency was the primary mediator of increased CD8^+^ T_Eff_/CD4^+^ T_Reg_ ratio. While COX2 inhibition limited tumor growth, NOS2 deficiency was critical for increased cure rates due to augmented antitumor B cell phenotypes associated with Nos2 depletion as reported earlier (24, 46). Importantly, these results were validated in the GEO dataset, where elevated CD8a/FOXP3 was predictive of improved survival in ER^–^ breast cancer patients (Figure 7C). Together, these results show that tumor COX2 and NOS2 have key roles in the regulation of immune polarization, as well as mobility and infiltration of CD8^+^ T cells into the tumor core.

### Spatially distinct immunosuppressive phenotypes.

**Figure 8:**
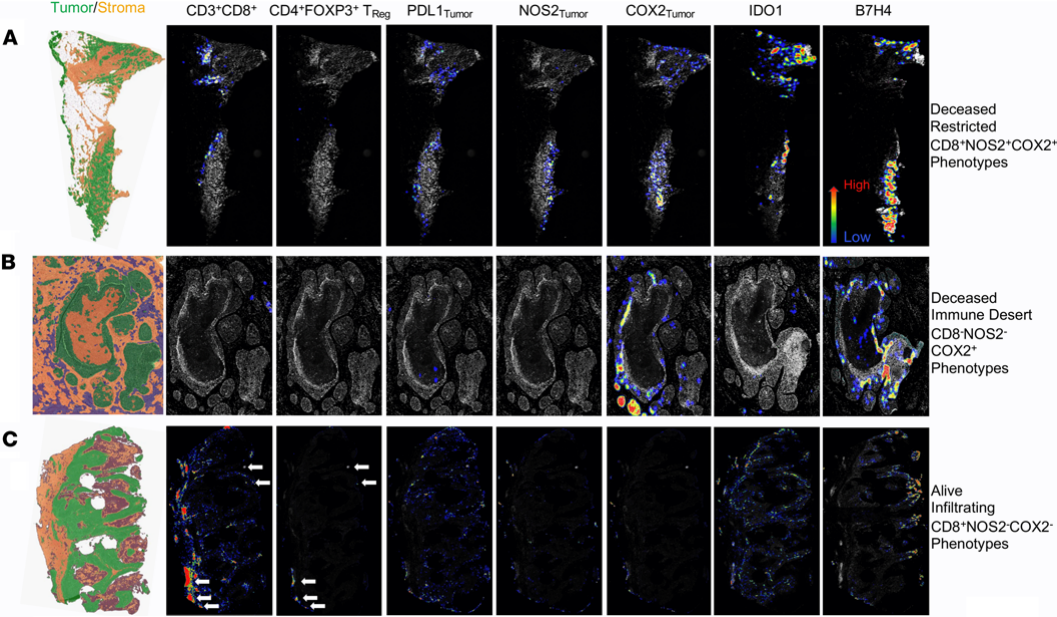
Density heatmap distributions of immunosuppressive T_Reg_, PDL1, IDO1, B7H4, and tumor NOS2 and COX2 phenotypes. Stroma (orange) as well as viable (green) and necrotic (purple) annotated tumor regions are shown in each panel (left). Density heatmap distributions with low-to-high scale are shown for CD3^+^CD8^+^ T cells, CD4^+^FOXP3^+^T_Reg_, PDL1_Tumor_, IDO1, B7H4, NOS2_Tumor_, and COX2_Tumor_ in NOS2/COX2-high tumors from deceased patients with (**A**) type I stroma-restricted CD8^+^ T cells with high NOS2 expression at the tumor margin and high COX2 expression deeper into tumor core and (**B**) type II immune deserts with abated CD8^+^ T cells, low NOS2 expression at the tumor margin, and high COX2 expression deeper into tumor core. (**C**) NOS2/COX2-low tumor from alive patient with high CD3^+^CD8^+^ T cell infiltration into the tumor. **B** and **C** show the same tumors as in Figure 6A (type III immune desert) and Figure 6C (alive patient).

Next, CD3^+^CD8^+^ T cell, CD4^+^FOXP3^+^ T_Reg_, PDL1_Tumor_, NOS2_Tumor_, COX2_Tumor_, IDO1, and B7H4 density heatmaps were spatially examined relative to stroma as well as viable and necrotic annotated tumor regions. Figure 8A shows stroma-restricted CD8^+^ T cell immune desert regions, as well as sparse CD4^+^FOXP3^+^ T_Reg_ and elevated PDL1_Tumor_ clusters in a deceased patient tumor with elevated tumor NOS2/COX2, which supports the existence of spatially distinct expression profiles. In addition, Figure 8B shows a deceased patient tumor with abated CD8^+^ T cells, sparse CD4^+^FOXP3^+^ T_Reg_ and PDL1_Tumor_, as well as low or abated NOS2_Tumor_ and high COX2_Tumor_ phenotypes. In contrast, surviving patients (Figure 8C) had low tumor NOS2/COX2 and high infiltrating CD8^+^ T_Eff_ cell density with low CD4^+^FOXP3^+^ T_Reg_ populations that were spatially aligned with CD8^+^ T_Eff_ cells (Figure 8C, white arrows). Interestingly, when normalized to NOS2_Tumor_ expression, elevated CD4^+^FOXP3^+^ T_Reg_ populations were statistically significant in alive patient tumors with low NOS2/COX2 tumor expression (Supplemental Figure 1). These results suggest that in the absence of tumor NOS2/COX2, the spatial alignment of CD4^+^FOXP3^+^ T_Reg_ populations with high-density CD8^+^ T_Eff_ cells and thus elevated intratumoral IFN-γ could indicate improved antitumor CD8^+^ T_Eff_ efficacy, which is supported by Figure 3B and Supplemental Figure 1B. Increased T_Regs_ and improved survival have been reported in some cancers (8, 47–51). In contrast, sparse CD4^+^FOXP3^+^ T_Regs_ in high NOS2/COX2-expressing tumors are associated with immune suppression and poor outcomes in this cohort. To further explore this observation, we examined RNA-Seq data for a potential Th1/T_Reg_ signature in 4T1 tumors from indomethacin-treated WT and Nos2^–^ mice. The results showed increased IL12RB1, TBX21, and IFN-γ in indomethacin-treated 4T1 tumors (Supplemental Figure 3A). These exploratory results support a Th1/T_Reg_ signature in indomethacin-treated 4T1 tumors; however, this information is not cell source specific.

In addition, low PDL1 levels were observed in low NOS2/COX2-expressing tumors from 5-year surviving patients (Figure 8C). Given that PDL1 can be induced by IFN-γ secreted from T_Eff_ cells, PDL1 expression could indicate improved treatment efficacy and tumor eradication in patients with high T_Eff_ infiltration (44). These 2 distinct profiles of tumors from deceased and living patients demonstrate the strong predictive value of tumor NOS2/COX2 expression with respect to an effective immune response and improved survival.

A previous report has shown distinct spatial localization of the immunosuppressive factors IDO1 and B7H4 in TNBC tumors (44). Earlier reports have shown that IDO1 was largely associated with the epithelial compartment of fully inflamed CD8^+^ T cell–enriched tumors associated with improved survival, while B7H4 was expressed in margin-restricted CD8^+^ T cell–low regions as well as immune deserts associated with a fibrotic signature and poor survival (44). Herein, survival analyses of IDO1 and B7H4 revealed no overall significance (data not shown). However, the ratio of NOS2s to IDO1 or B7H4 was significantly elevated in tumors from deceased patients (Supplemental Figure 3B). Given that NOS2, IDO1, and B7H4 are induced by IFN-γ (52), this may reflect T_Eff_/IFN-γ spatial and/or functional relationships. Previous studies have identified IDO1 as a favorable prognostic indicator for ER^+^ but not ER^–^ breast cancer (53). Herein, tumor NOS2 and IDO1 are inversely correlated with ER^–^ breast cancer survival (Supplemental Figure 3B). High NOS2/COX2-expressing tumors exhibited increased IDO1 clustering that was more closely associated with tumor margins proximal to elevated NOS2_Tumor_ regions (Figure 8A), while low expression was observed in immune desert regions in deceased patient tumors (Figure 8B). In contrast, high CD8^+^ T_Eff_ infiltration in low NOS2/COX2-expressing tumors was associated with evenly distributed IDO1 expression spatially localized within tumor epithelia (Figure 8C), which is consistent with the findings of Gruosso et al. (44). In addition, the spatial examination of B7H4 revealed higher B7H4 clustering associated with stroma/margin-restricted CD8^+^ T cells and immune desert regions in tumors from deceased patients (Figure 8, A and B, respectively), where immune deserts contained both B7H4 and COX2. Thus, increased IDO1 clustering is associated with inflamed regions containing stroma-restricted CD8^+^ T cells proximal to elevated tumor NOS2 while the immune desert contains predominately COX2 and B7H4 in tumors from deceased patients. In contrast, lower IDO1 clustering is more evenly distributed in tumors from surviving patients with low NOS2/COX2 expression. These results implicate a landscape of distinct immunosuppressive cellular phenotypes that is driven by tumor NOS2 and COX2 expression.

## Discussion

Poor outcomes for cancer patients are associated with metastasis, chemoresistance, and immune suppression. Immunosuppressive TMEs limit therapeutic efficacies, thus warranting interventions that target immune-suppressive pathways. Our study implicates tumor NOS2 and COX2 as key targets for limited immune suppression and improved antitumor immune response. IFN-γ produced from stroma-restricted T_Eff_ cells is crucial for the induction of tumor NOS2/COX2 expression in TNBC (12), where the resultant NO/PGE2 production inhibits B cell activation and T_Eff_ cell antitumor function, respectively (54–56). This dichotomy represents a classic negative feedback mechanism, where IFN-γ from activated T_Eff_ cells increases tumor NOS2 and COX2 expression, dampening the Th1 antitumor response. The specific spatial orientation of T_Eff_/NOS2/COX2 provides a key determinant of outcome that shapes the tumor immune microenvironment. Patients with positive outcomes have low or sporadic tumor NOS2/COX2 expression and increased CD8^+^ T_Eff_ cells that penetrate deep into the tumor core (40). In contrast, patients with stroma- or margin-restricted CD8^+^ T_Eff_ cells and elevated tumor NOS2/COX2 are associated with abated CD8^+^ T_Eff_ penetration and increased tumor invasion and metastasis (38, 40, 42). Thus, the spatial relationship between tumor NOS2/COX2 expression and CD8^+^ T_Eff_ cells is a key predictive factor of antitumor immune response.

Herein, we identify 3 distinct types of CD8^+^ T cell exclusion from the tumor core associated with tumor NOS2 and COX2 expression that correlate with poor survival: type I, restricted inflamed, with stromal restricted CD8^+^ T cell inflamed regions and elevated tumor NOS2 and COX2 expression; type II, developing immune deserts with limited CD8^+^ T cell penetration, high tumor COX2, and abated tumor NOS2 expression; and type III, mature immune deserts lacking both CD8^+^ T cells and tumor NOS2, as well as low tumor COX2, and elevated B7H4 expression. Another feature that distinguishes type I and II from type III immune deserts is that type I and II immune deserts exhibit proximal lymphoid aggregates to the tumor (40), which are not observed in type III mature immune deserts. Although these are distinct regional phenotypes, it is not uncommon to observe all of them in the same tumor, which may be linked during disease progression.

The exclusion of CD8^+^ T cells from the tumor prevents the cell-to-cell contact necessary for T_Eff_ cell release of cytolytic effector molecules, including IFN-γ and granzyme B. This immune-specific cell interaction targets tumor and virally infected cells, limiting damage to normal tissue in contrast with the nonspecific killing of rapidly growing cells that occurs with conventional therapies (57–60). Thus, interventions that enhance the tumoricidal activity and number of infiltrating T cells are therapeutically beneficial. Previous TNBC studies have shown that CD8^+^ T cell restriction was predictive of poor survival, consistent with other reports in various cancers (61–64). Importantly, tumor NOS2/COX2 expression is a key determining factor in the penetration versus restriction of lymphoid aggregates (24, 40, 46).

While NOS2 and COX2 affect the whole tumor landscape, they also influence specific tumor regions, including large tumor nests, lymphoid aggregates, tumor edge, tumor core, and tumor satellites, which are affected differently by the spatial configuration and distribution of T_Eff_/NOS2/COX2 phenotypes (40). Also, each region has a distinct immunosuppressive mechanism. For example, the core of larger tumors is predominated by COX2 and B7H4, while the inflamed edge is NOS2^+^/COX2^+^. Within lymphoid aggregates, immune cell–based immunosuppression predominates, such as T_Reg_ and PDL1_Macrophage_. The specific regions of immune suppression relative to T_Eff_/NOS2/COX2 spatial configuration provide insight into the role of tumor NOS2 and COX2 during disease progression.

Given that the spatially defined immune-suppressive niches share common mechanisms, the complementary use of immune-activating therapeutics could have synergistic effects targeting these different regions. In the type I configuration, the increased restricted lymphoid cells reside primarily in the lymphoid aggregates, where immune polarization of this region can impact survival. The survival comparisons of T_Eff_ cells with other markers in these regions showed 2 predictive phenotypes: CD4^+^ T_Reg_ and PDL1_Macrophage_. The 4T1 model supports this observation, where systemic NOS2 depletion or COX2 inhibition increased the ratios of CD8^+^ T_Eff_/CD4^+^ T_Reg_ and CD8^+^ T_Eff_/PDL1_Macrophage_ that were associated with improved survival. In addition, analysis of myeloid populations revealed increased CD11c^+^CD8^+^ antigen-presenting DCs in NOS2/COX2-low tumors from alive patients and indomethacin-treated 4T1 tumor-bearing mice, as well as increased F4/80^+^MHC II^+^ antigen-presenting macrophages in the indomethacin-treated 4T1 tumor-bearing mice (Supplemental Figure 4). These results further support the hypothesis that tumor NOS2 and COX2 are predictive of lymphoid aggregate immune polarization.

High T_Reg_ cells and low CD8^+^ T cells in the TME of many tumors are associated with poor survival (65, 66). Feed-forward production of T_Reg_ phenotypes is induced by TGF-β, IFN-γ, and IL-4 (67–69), which promotes IDO1 and kynurenine production that further increases T_Regs_ along with numerous immunosuppressive cytokines, including IL-10 and TGF-β. Both PGE2 and NO can increase immunosuppressive mediators of T_Regs_, such as IL-10. T_Regs_ suppress T_Eff_ cell function through direct PGE2 stimulation of IL-10. Because NOS2 and COX2 promote cytokine release and activation of IL-10 and TGF-β, NOS2 and COX2 tumor expression could complement one another regionally to maintain immune suppression.

Interestingly, we observed T_Reg_ clusters that aligned with high CD8^+^ T cell densities in tumors from alive patients with low NOS2/COX2 tumor expression (Figure 8C). Under the influence of IL-12, FOXP3^+^ T_Regs_ can differentiate to Th1-like effector cells that express the Th1 lineage–specific T-bet transcription factor (TBX21). In addition to IL-12, this differentiation process is also facilitated by IFN-γ, which is secreted by CD8^+^ T_Eff_ cells. These cells acquire a Tbet^+^FOXP3^+^IFN-γ^+^ phenotype with limited immunosuppressive but enhanced pro-inflammatory capabilities, which is consistent with our exploratory analysis in Supplemental Figure 3A (70).

While PDL1_Tumor_ expression can be located at the tumor-stromal interface, PDL1 is also expressed on tumor-associated macrophages (TAMs) that reside primarily in lymphoid aggregates. PDL1_Macrophage_ expression can be induced by IFN-γ, TNF-α, and IL-6 as part of negative feedback regulation (71, 72). TNF-α and IL-6 activate the NF-κB and STAT3 signaling pathway to regulate PDL1 expression on M2 macrophages (71–74), where increased PDL1_Macrophage_ expression is involved in the developing TAM phenotypes (72, 75). PDL1_Macrophage_ has been shown to inhibit CD8^+^ T_Eff_ cell function in collaboration with IDO1 or granulin (76–80). The IDO1 density heatmaps show a strong association with high CD3^+^CD8^+^ regions, suggesting that an increase in this M2 macrophage phenotype relative to CD8^+^ T_Eff_ cells limits antitumor CD8^+^ T cell function. Thus, the increased density of immunosuppressive cytokines and proximity to IDO1 clusters suggests that poor clinical outcomes are due, in part, to the induction of the M2 immunosuppressive phenotypes that limit the antitumor function of neighboring lymphoid aggregates.

Type I restricted inflamed tumors are observed in high NOS2/COX2-expressing tumors. The relative positioning of tumor NOS2 and COX2 to CD8^+^ T_Eff_ cells suggests an immune-suppressive progression from inflamed foci to immune desert regions devoid of lymphocytes. Increased NOS2-derived NO promotes metastasis, where higher clustering of NOS2^+^ tumor cells leads to increased oncogenic pathway signaling and immune-modulatory proteins, including IL-1, IL-6, IL-8, and TNF-α (12, 81). Regions of elevated tumor NOS2/COX2 would be expected to increase cancer cell stemness (40), thus leading to therapy resistance while causing increased activation of TGF-β and IL-10. The diffusion of these immunosuppressive factors would counter Th1 inflammation, favoring more immunosuppression. Thus, the type I configuration (Figure 6A) is highly inflamed, with distinct areas that fuel complementary immune-suppressive mechanisms. Therefore, the interaction of lymphoid aggregates at the tumor edge leads to immune suppression and increases chemoresistance and metastasis, the hallmark of poor outcomes.

Type II and type III immune deserts represent regions with CD8^–^NOS2^–^COX2^+/–^ phenotypes (40). These are immunologically cold tumor regions, where type II immune deserts have lymphoid aggregates proximal (<0.5 mm) to the tumor edge while type III immune deserts do not. High COX2 rather than NOS2 suggests the importance of PGE2 from the tumor in maintaining the exclusion of CD8^+^ T cells (40). The lack of both tumor infiltrating lymphocyte (TIL) aggregates and NOS2^+^ tumor edges observed in the type III immune desert regions is also associated with lower COX2 expression and is consistent with NOS2/COX2 feed-forward signaling (25). In addition, abundant B7H4 expression was observed in type III immune desert regions. Thus, these immune desert regions could mechanistically promote therapeutic resistance due to abated CD8^+^ T_Eff_ cell infiltration (82, 83). The 4T1 model produces these type II/III immune desert regions with features that resemble human tumors. The addition of sublethal radiation damage simply augments the marginal immunosuppressed populations, despite cell injury (46). However, NOS2 and COX2 inhibition activates CD8^+^ T_Eff_ cell function, leading to increased granzyme B (24, 40, 46). The enrichment of type II/III immune desert phenotypes through damage induced by treatment or tumor stressors, including hypoxia and/or ischemia/reperfusion, can recruit lymphocytes that produce IFN-γ and cytokines. However, they also induce a robust NOS2/COX2 immune-suppressive response, thus leading to poor clinical outcome (12, 40). Several studies show that more advanced tumors have poorer responses and reduced TIL penetration into the tumor core (84–87). This observation would reflect a fortified tumor barrier where preferential activation of NOS2/COX2 dramatically limits antitumor immune response (24, 40).

In more advanced tumors such as chemoresistant breast cancer, the combination of tumor cell chemoresistance and immunosuppression results in poor outcomes. A recent study of chemoresistant TNBC patients treated with a pan-NOS inhibitor and low-dose aspirin improved survival with more than 88% response in locally advanced tumors, which was associated with increased CD8^+^ T cells, M1 macrophages, N1 neutrophils, and B cell populations (27). Remarkably similar changes were observed in the 4T1 model, where systemic NOS2 depletion and indomethacin treatment led to increased CD8^+^ T cell infiltration, N1 neutrophils, and active B cells, which translated to increased cures in 4T1 tumor-bearing mice (24). In this model, indomethacin alone only targeted the T cells without cure, which required augmented B cells associated with systemic NOS2 depletion (24). Together, this work demonstrates that targeting NOS2 and COX2 together augments antitumor immune responses of aggressive ER^–^ breast tumors.

**Figure 9:**
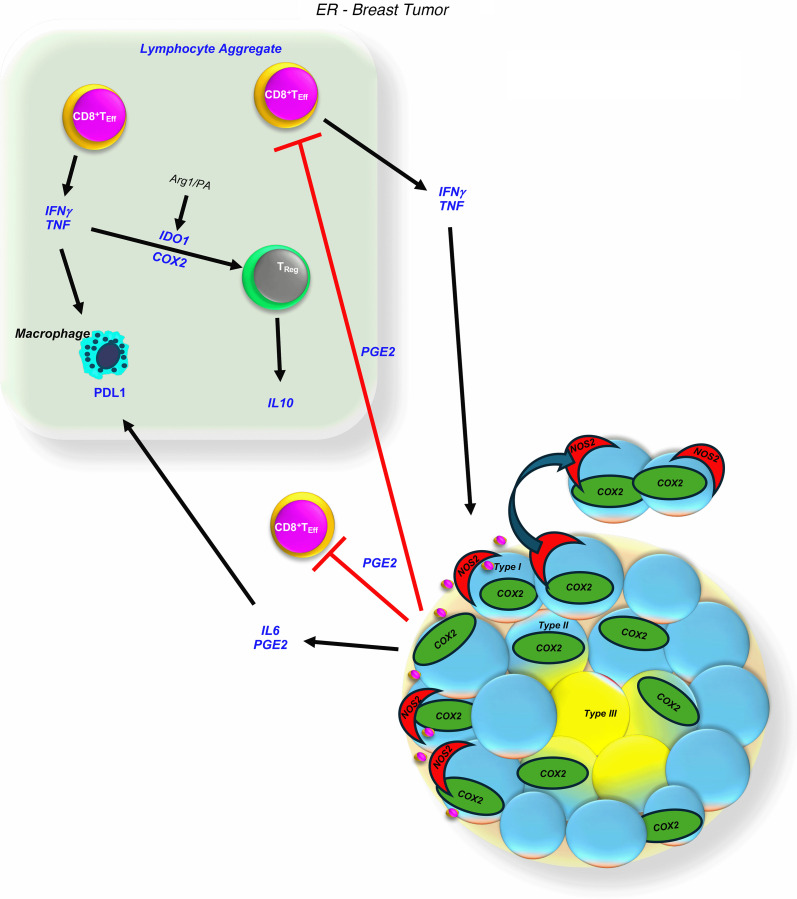
Summary of immunosuppressive signaling progression. Stroma-restricted CD8^+^ T_Eff_ cells secrete IFN-γ/TNF-α that induces tumor NOS2 and COX2 expression near tumor margin, leading to type I T_Eff_ restricted regions. Tumor COX2-expressing type II immune deserts with limited T_Eff_ infiltration progress to type III immune desert regions with abated NOS2 and COX2 tumor expression. IDO1 and PDL1_Macrophage_ are expressed in tumor stroma along with increased T_Reg_ populations in lymphoid aggregates.

NOS2 and COX2 tumor expression shapes the immune landscape and mediates the transition from inflamed regions to immune deserts. It is important to appreciate the distinct regional mechanisms of immune suppression resulting from the differential expression of NOS2 and COX2. The formation of an immune desert and the subsequent resistance to treatment linked to tumor NOS2/COX2 induction indicates that novel treatments targeting these proteins may be therapeutically beneficial for the treatment of aggressive drug-resistant tumors. NOS2 and COX2 augment multiple immunosuppressive pathways and increase the severity of immunosuppression (Figure 9). Determining the immunosuppressive phenotypes of distinct regions of the tumor landscape provides insight into multiple local mechanisms affecting tumor survival. The introduction of checkpoint inhibitors has provided a new approach for the treatment of various malignancies. In breast cancer, however, checkpoint inhibitors have been less effective (88–90). Immune-suppressive factors that reduce the efficacy of many cancer treatments present a major obstacle to successful therapy. According to the data presented herein, restriction and even elimination of CD8^+^ T_Eff_ cells in NOS2/COX2-high tumors suggests that NO and PGE2 play an essential role in immune suppression, where agents that control their activity offer an important clinical tool for augmented antitumor immune response.

## Methods

Further information can be found in Supplemental Methods.

### Sex as a biological variable.

Pearson’s correlation coefficients for the incidence rates of female versus male breast cancer have been reported (91). Male breast cancer rates were generally less than 1 per 100,000 person years, in contrast with the much higher rates of female breast cancer of 122. The differences in both incidence rates and time trends between males and females may reflect sex differences in underlying risk factors, including differences in ducts and lobules and the absence of p53 mutation (92). While most males are ER^+^ and ductal carcinoma in situ represents 10% of male breast cancers, ER^–^ and TNBC are less frequent with poorer prognosis due to higher histopathological grade (93). Given these low occurrences in males, female patients with ER^–^ tumors were examined for the effects of tumor NOS2 and COX2 expression on radiation therapeutic efficacy. This work adheres to Animal Research: Reporting of In Vivo Experiments guidelines.

### Statistics.

Experiments were assayed in triplicate unless otherwise stated. Mann Whitney 2-tailed analyses were used, except in Figure 7A (CD8^+^ T_Eff_/PDL1_Macrophage_) where a 1-tailed analysis was used, to assess statistical significance using GraphPad Prism software (version 9; RRID: SCR_002798). In some cases, outliers were excluded based upon mathematical analysis using Prism software ROUT method. Image analyses are reported as mean ± SEM, and Mann-Whitney 2-tailed analyses were used when appropriate to determine significance. Linear analyses and Pearson’s correlations were also conducted to determine significant correlations between protein expressions using Prism software (10.1.2). Significance was considered at *P* ≤ 0.05.

### Study approval.

Studies were conducted in accordance with recognized *Guiding Principles for Ethical Research* (94) following the Declaration of Helsinki. Studies were approved by institutional review boards and in accordance with assurance filed with and approved by the US Department of Health and Human Services (OHSR no. 2248) and University of Maryland, Baltimore, Maryland (protocol no. 0298229), as well as by the Institutional Review Board of Emory University for human studies. Written informed consent was obtained from all patients. Mouse studies were approved in IBC 2023-42 and ACUC 21-109. MDA-MB-231 cells were obtained from ATCC and approved for use in biosafety protocol IBC 2023-42.

### Data availability.

Public repository data from combined GEO datasets (https://www.ncbi.nlm.nih.gov/geo/; *n* = 796) generated using https://kmplot.com/analysis are available in the provided links. Supporting data values are provided online in the Supporting Data Values XLS file.

## Author contributions

LAR performed data analysis and wrote and edited the manuscript. RYSC performed data analysis. WFH performed data analysis. MP performed data analysis. ALG performed data analysis. ELF performed data analysis. RLM performed data analysis. ALW performed data analysis. FI performed data analysis. LLC performed data analysis. DB performed experiments. EFE performed data analysis. MCR provided resources. STCW provided resources. SL provided resources. SAG provided resources. MPV edited manuscript. DWM edited the manuscript. XL provided resources. SKA edited manuscript. NP edited manuscript. SMH performed data analysis. SA provided resources. TRB provided resources. JCC provided resources. SJL provided resources. DAW conceived the idea, provided resources, and wrote the manuscript. The order of co–first authors was decided on after discussion among the authors.

## Supplementary Material

Supplemental data

Unedited blot and gel images

Supporting data values

## Figures and Tables

**Table 1:**
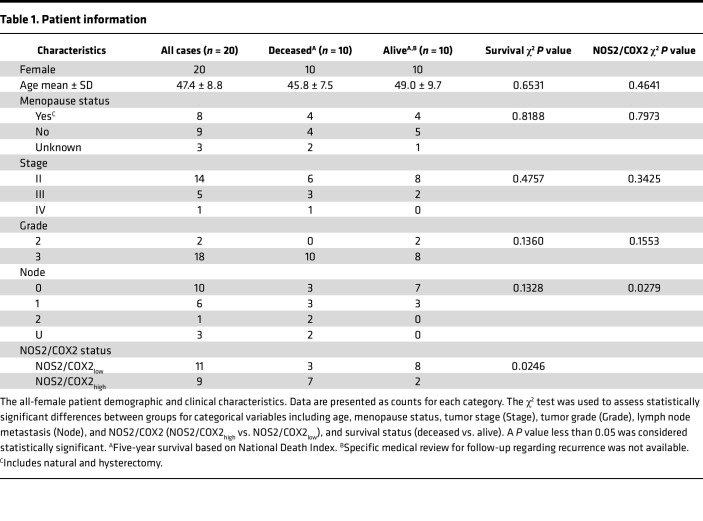
Patient information

## Version 1 07/15/2025

In-Press Preview

## Version 2 08/22/2025

Electronic publication
