# Supplementary material for: Elevated tumor NOS2/COX2 promotes immunosuppressive phenotypes associated with poor survival in ER– breast cancer
Source: JCI Insight. 2025 Jul 15;10(16):e193091. doi: 10.1172/jci.insight.193091 (PMC12406732; doi:10.1172/jci.insight.193091)
Supplement: Supplemental data [file jciinsight-10-193091-s196.pdf]

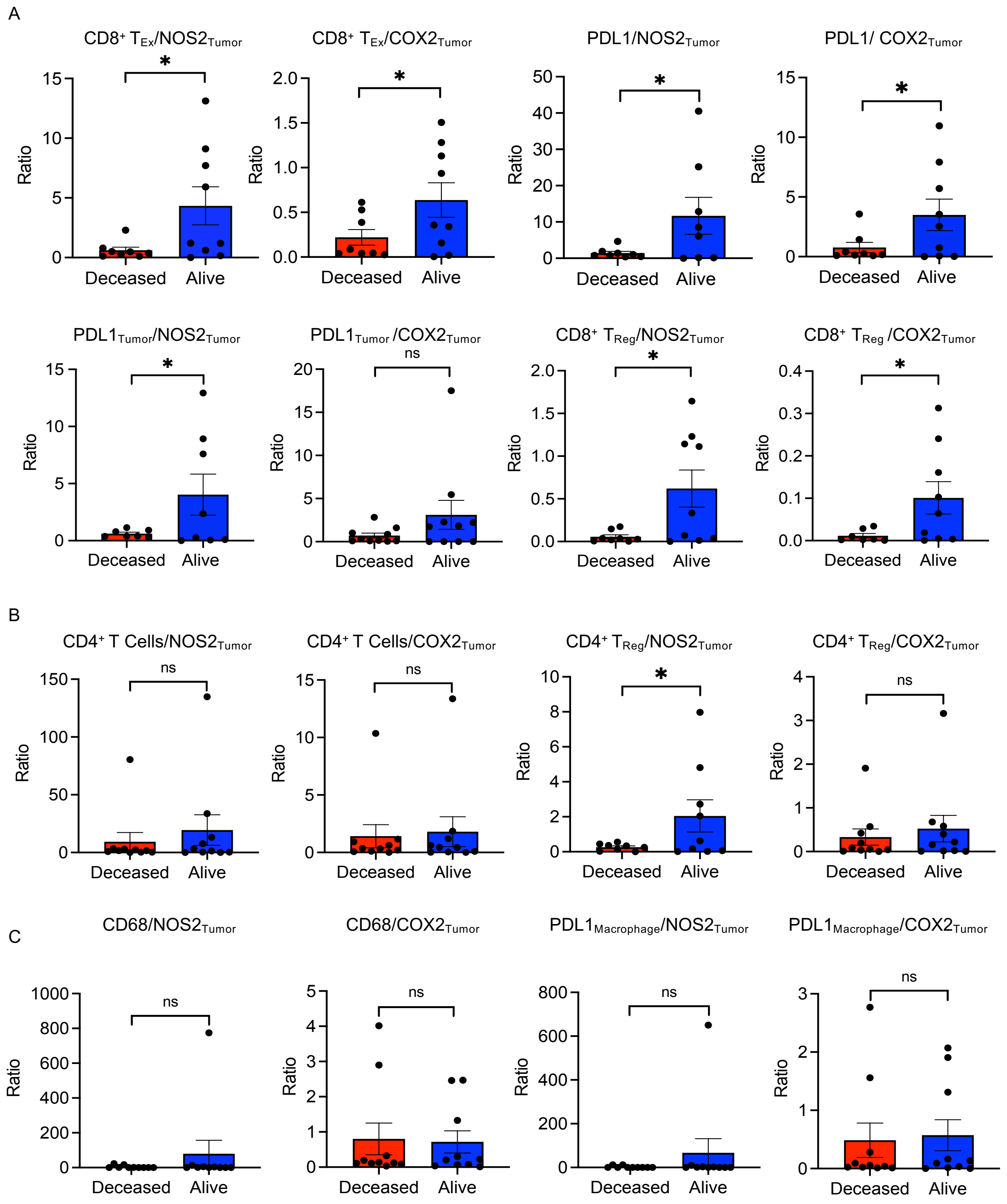

Supplemental Figure 1

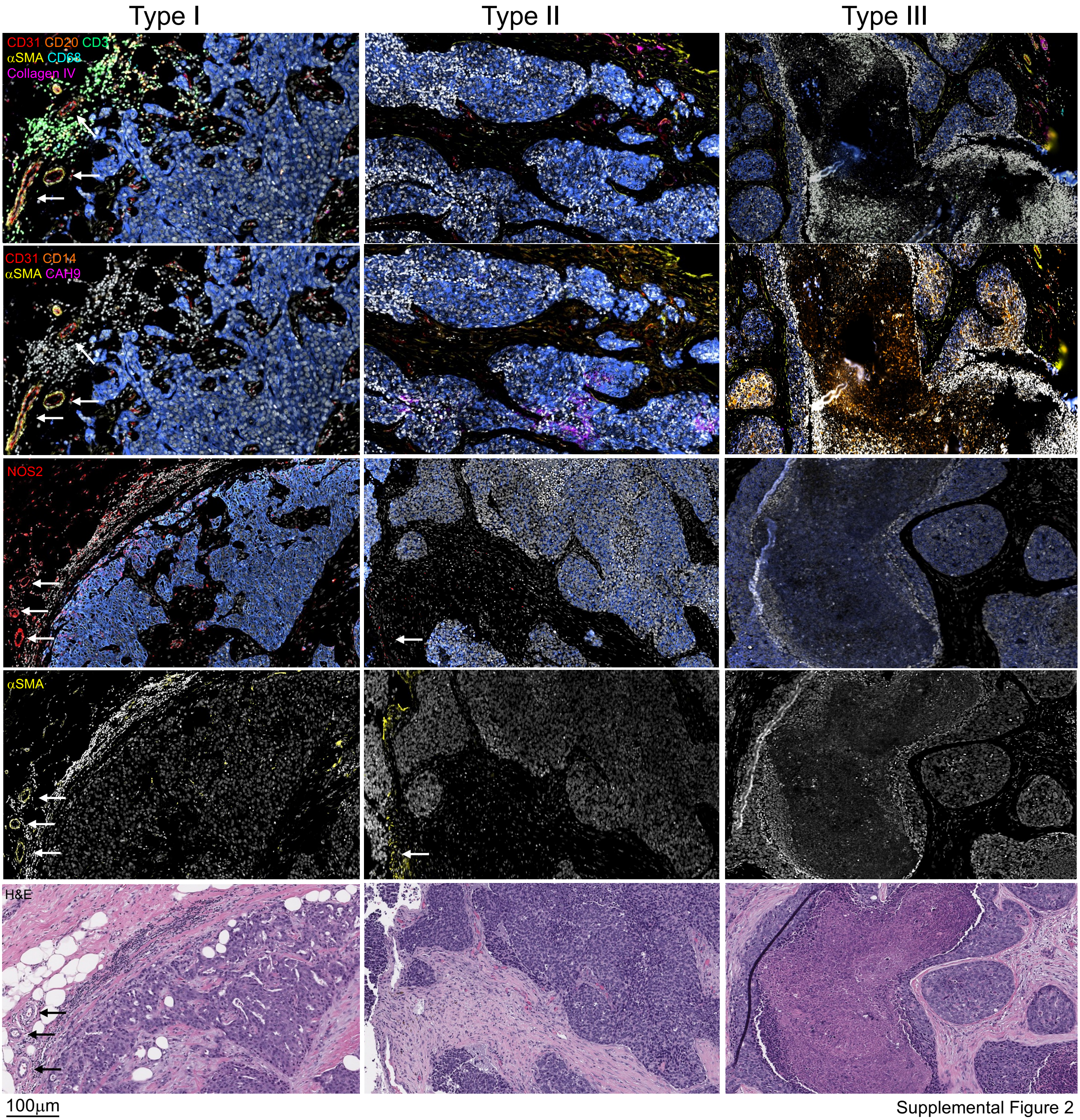

Supplemental Figure 2

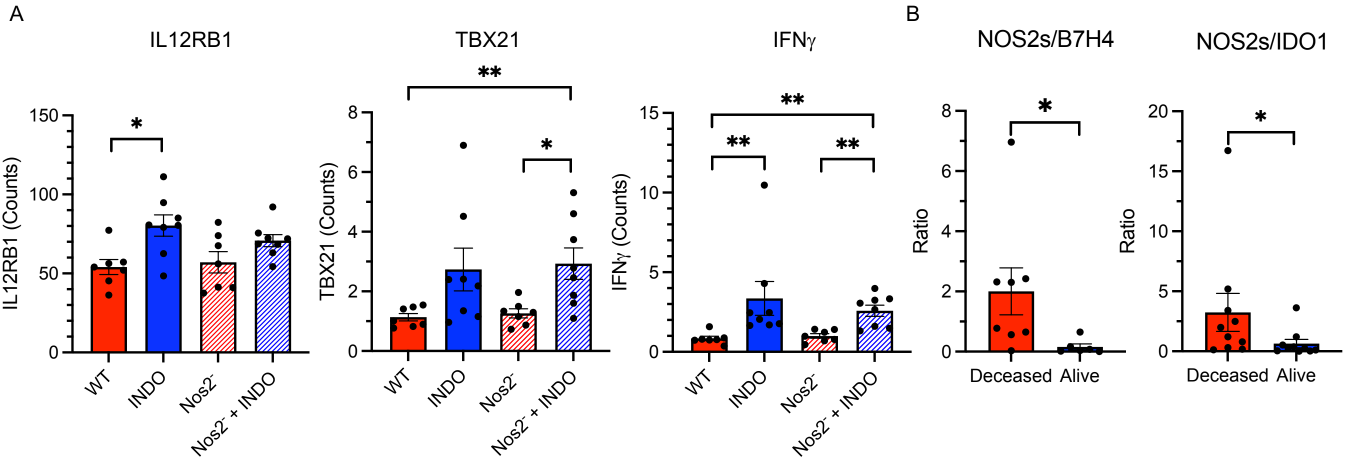

Supplemental Figure 3

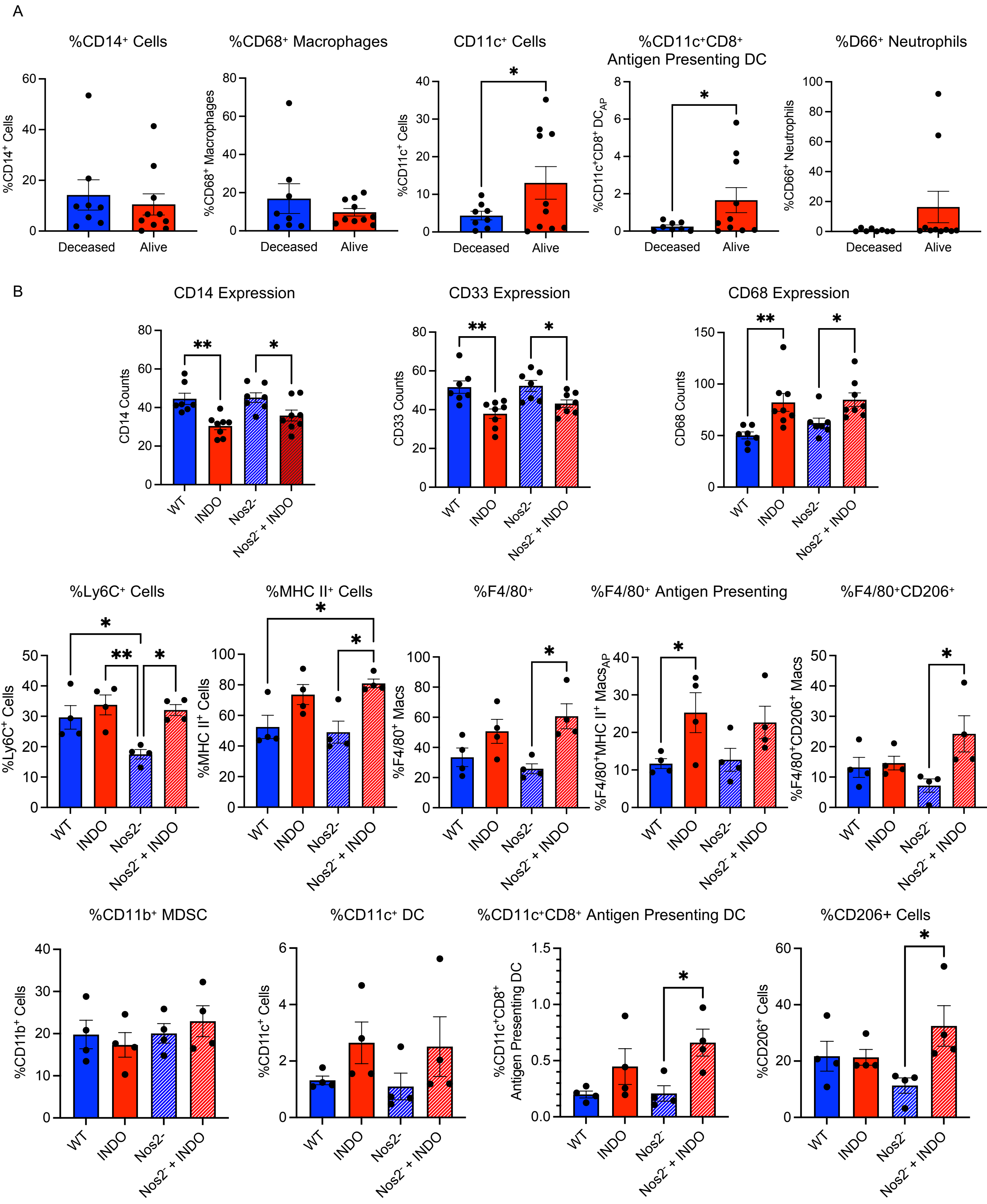

Supplemental Figure 4

Supplemental Information

Tissue Collection and Immunohistochemical Analysis of Patient Tumor Sections

A retrospective study based upon a historical collection of tumor specimens obtained from patients with breast cancer recruited at the University of Maryland (UMD) Medical Center, the Baltimore Veterans Affairs Medical Center, Union Memorial Hospital, Mercy Medical Center, and the Sinai Hospital in Baltimore between 1993 and 2003 is reported. Written informed consent was obtained from all patients. Clinical and pathologic information was obtained from medical records and pathology reports and associated with unique patient identifier numbers. Disease staging was performed according to the tumor node–metastasis system of the American Joint Committee on Cancer/Union Internationale Contre le Cancer (15). The Nottingham system was used to determine the tumor grade (16). There was no linkage to personal identifiers for these patients, and attrition information was not available. Each tumor sample was identified by an accession number for blinding purposes. Breast tumor NOS2 and COX2 expression was analyzed previously by IHC using 1: 250 diluted NOS2 antibody and 1:50 diluted COX2 antibody [no. 610328 (RRID: AB_397718) and 610204 (RRID: AB_397603), respectively, BD Biosciences] and scored by a pathologist (4, 17). For NOS2 staining, a combination score of intensity and distribution was used to categorize the IHC NOS2 stains where intensity received a score of 0 to 3 if the staining was negative, weak, moderate, or strong, respectively. The NOS2 distribution received scores of 0 to 4 for distributions <10%, 10% to 30%, >30 to 50%, >50 to 80%, and >80% positive cells (4). For COX2 staining, scores of negative to weak (1–2) or moderate to strong (3–4) were categorized as low or high, respectively (17). Herein, the tumor immune microenvironment was examined in NOS2Hi/COX2HI (n = 10) versus NOS2Lo/COX2Lo (n = 10) expressing ER- tumors, which included tumors with TNBC (16) and HER2-positive (5) status and correlated with 5-year survival. Of the 21 ER- samples examined; nine patients received presurgery/neoadjuvant treatment prior to tumor resection. However, at the time of tumor resection, which was performed after a recovery period, the influence of neoadjuvant therapy should have disappeared. We examined tumors that were either NOS2Hi/COX2Hi (11) or NOS2Lo/COX2Lo (10) in tumor expression. Some tumors were NOS2Hi/COX2Lo or NOS2Lo/COX2Hi, and these samples were excluded. In addition, some tumors were excluded based upon disease-specific survival at 5 years postdiagnosis; some patients died of unknown or other causes. Several blocks were no longer available due to usage/tissue exhaustion and could not be examined. Specific medical review for follow-up

regarding recurrence was not available; the National Death Index was searched in 2006, 2008, 2010, and 2020 for survival follow-up. Since the 5-year mark, four women have succumbed with malignant neoplasm of breast at 6.5, 8.4, 10.4, and 24.3 years postdiagnosis or surgery. Information of active disease in surviving patients at the 5-year mark was not available.

The influence of tumor NOS2 and COX2 expression on 5-year disease specific survival is reported. All tumors were evaluated and annotated by a pathologist for viable and necrotic tumor, as well as tumor stroma for the localization of biomarker expression. NOS2 and COX2 expressions were analyzed by multiplex fluorescent staining performed on the Leica Biosystems BOND RX Autostainer 21.2821 ST5010 using the BOND Polymer Refine Kit (Leica Biosystems DS9800), with omission of the Post Primary reagent, 3, 3'-diaminobenzidine (DAB), and hematoxylin. After antigen retrieval with EDTA (BOND Epitope Retrieval 2), sections were incubated for 30 minutes with COX2 [Cell Signaling Technology, no. 12282 (RRID: AB_2571726), 1:100], followed by the polymer reagent and Opal Fluorophore 520 (Akoya Biosciences). The COX2 antibody complex was stripped by heating with BOND Epitope Retrieval 2. Sections were then incubated for 30 minutes with NOS2 antibody [Abcam no. ab15323 (RRID: AB_301857), 1:50], followed by the polymer reagent and Opal Fluorophore 690. The NOS2 antibody complex was stripped by heating with BOND Epitope Retrieval 2 and then stained for a third target, either CD8 [Abcam no. 101500 (RRID: AB_10710024), 1:100], IFNγ [Abcam no. 231036 (RRID: AB_2941995), 1:200], IDO1 [ThermoFisher no. A700-031 (RRID: AB_2765300)], and B7H4 [Cell Signaling no. 14572 (RRID: AB_2750878)] followed by the polymer reagent or Opal Fluorophore 570. Sections were stained with 4',6-diamidino-2-phenylindole (DAPI) and coverslipped with ProLong Gold Anti-Fade Reagent (Invitrogen). Images were captured using the Aperio ScanScope FL whole slide scanner (Leica Biosystems). The original IHC previously reported (4, 17) and fluorescent NOS2/COX2 staining results were generally consistent.

Formalin-fixed paraffin embedded (FFPE) tissue sectioned at 4 μm and mounted on SuperFrost Plus slides were stained with a FixVUE Immuno-8™ Kit [formerly referred to as UltiMapper® kits (Ultivue Inc., Cambridge, MA), USA; (RRID: AB_3665705)] using the antibody conjugated DNA-barcoded multiplexed immunofluorescence (mIF) method to visualize CD3, CD4, CD8, FoxP3, PD-1, and PD-L1 (1). This kit includes the required buffers and reagents to run the assays: antibody diluent, pre-amplification mix, amplification enzyme and buffer, fluorescent probes and corresponding buffer, and nuclear counterstain reagent. Hematoxylin and

Eosin (H&E) and mIF staining was performed using the Leica Biosystems BOND RX Autostainer. Before performing the mIF staining, FFPE tissue sections were baked vertically at 60-65 °C for 30 min to remove excess paraffin prior to loading on the BOND RX. The BOND RX was used to stain the slides with the recommended FixVUE (UltiMapper) protocol. During assay setup, the reagents from the kit were prepared and loaded onto the Autostainer in Leica Titration containers. Solutions for epitope retrieval (ER2, Leica Biosystems cat# AR9640), BOND Wash (Leica Biosystems cat# AR9590), along with all other BOND RX bulk reagents were purchased from Leica). During this assay, the samples were first incubated with a mixture of all 4 antibody conjugates, next the DNA barcodes of each target were simultaneously amplified to improve the sensitivity of the assay. Fluorescent probes conjugated with complementary DNA barcodes were then added to the sample to bind and label the targets; A gentle signal removal step was used to remove the fluorescent probes of the markers after imaging. Prior to imaging, the stained slides were mounted in ProLong Gold Anti-Fade Mountant (Thermo Fisher Scientific, Waltham, MA, cat# P36965) and coverslipped (Fisherbrand Cover Glass 22 x 40mm, #1.5). Digital immunofluorescence images were scanned at 20X magnification. Images were co-registered and stacked with Ultivue UltiStacker software. The digital images were then analyzed using HALO image analysis platform (109).

Previous studies indicate that NOS2 fluorescence intensity thresholds of total tumor intensity are associated with a worse prognosis. Here, the intensity levels of NOS2 and COX2 at the single-cell level were determined using thresholds of strong, medium, and weak as previously determined (12, 24). Strong signal intensity of tumor NOS2 ($NOS2_s$) was approximately 6-x higher in tumors from Deceased vs Alive patients as compared to $NOS2_w$ (weak) or $NOS2_{all}$ (total NOS2) signal intensities, which were 2-x elevated (Fig. Sup. 1A). In contrast, the maximum difference and statical significance of COX2 did not change over the thresholds. Thus, the thresholds for $NOS2_s$ and $NOS2_{all}$ and $COX2_{all}$ were used. The fluorescence and original IHC findings were generally consistent (25, 54, 55).

SUMAP Neighborhood Clustering Analysis

The spatial Uniform Manifold Approximation and Projection for Dimension Reduction (S-UMAP) (61) and neighborhood analysis was used to identify cellular neighborhoods correlated with clinical outcome. Single cell neighborhood density profiles ($d_{pheno}(r)$) constructed of nine phenotypes ($CD4^+FOXP3^+$ $CD8^+FOXP3^+$, $CD8^+CD3^+PD1^+$, tumor $NOS2^+$, tumor $COX2^+$, tumor

PDL1$^+$, CD68$^+$PDL1$^+$, CD68$^+$COX2$^+$, and Other) at 25, 50, 100, 150, 200 μm distances to identify similar neighborhoods. This analysis provided a single cell neighborhood profile with a 45-value descriptor of each cell neighborhood (nine phenotypes by five distance bands). The neighborhood profiles of all tumors were dimensionally reduced via UMAP and plotted as a 2D histogram, and K-means clustering was used to identify clusters of similar neighborhoods. For each cluster, the ratio of the percentage of deceased cell neighborhoods contributing to that cluster over the percentage of alive cell neighborhoods contributing to that cluster was calculated. The clusters with the greatest and the lowest ratio values, representing neighborhoods over-represented in deceased patient samples (C_D cluster 13 in this analysis) and alive patient samples (C_A, cluster 11 in this analysis), respectively, were identified. Next, the relative density profiles of each phenotype between the over-represented clusters, $d'_{pheno}(r)$, was calculated by dividing the average density profile of that phenotype from the C_D and C_A clusters:

$$d'_{pheno}(r) = d_{pheno}(r, C_D)/d_{pheno}(r, C_A)$$

Analyses were performed using MATLAB custom software and the UMAP library (**https://www.mathworks.com/matlabcentral/fileexchange/71902**), (MATLAB Central File Exchange RRID: SCR_001622]; (110). Neighborhood profiles are plotted as mean ± SEM.

The Gene Expression Omnibus (GEO) Validation

Array-based breast cancer survival plots of compiled databases can be generated using the https://kmplot.com/analysis portal, which consolidates different publicly accessible repositories into one single platform as reported by Gyorffy (59). This tool was used to validate the predictive power of the NOS2/IFNG and COX2/IFNG relationships in compiled ER- breast tumor datasets found in the GEO repository (https://www.ncbi.nlm.nih.gov/geo/) [RRID:SCR_005012]. In addition, these relationships were validated using gene expression of our ER- breast tumor cohort (GSE37751) also found in (https://www.ncbi.nlm.nih.gov/geo/) [RRID:SCR_005012]. The R software (https://www.r-project.org/ version 4.4.3) was used to extract gene expression data from ER- samples for subsequent analysis. Briefly, NOS2, COX2, IFNG, CD8A, and FOXP3 gene expression and associated survival data were extracted and processed together. The data sets processed for NOS2/IFNG and COX2/IFNG (Fig. 2D) as well as CD8A/FOXP3 (Fig. 7C) relationships by dividing the data into subsets according to the median gene expression values of these relationships. High (red) and low (black) ratios dichotomized at the median were calculated. The associated survival was exported to Prism (v10) and probability of survival was plotted. The

P values were determined using Log-rank (Mantel-Cox) test and hazard ratios were calculated using Mantel-Haenszel test.

PD1 Inhibitor Clinical Trial

Formalin fixed paraffin embedded (FFPE) biopsies from 18 deidentified patients treated with the neoadjuvant KEYNOTE 522 (K522) regimen at Emory University were included. All hematoxylin and eosin (H&E) stained biopsy slides were evaluated by a board-certified breast pathologist. Response to neoadjuvant K522 was evaluated at the time of surgery and classified as pathologic complete response (pCR n = 8) or non-pCR (n = 10), where pCR was defined as no invasive carcinoma in the breast and axillary lymph nodes at the time of surgery. Follow-up data including metastasis, local recurrence and overall survival were retrieved from patients' charts. Other clinical information including initial clinical staging, race, and body mass index (BMI) was also retrieved from patients' charts. Tumor NOS2, COX2, and CD8 were evaluated with OPAL as described above.

***In vivo* Studies**

Animal care was provided at the NCI-Frederick Animal Facility according to procedures outlined in the Guide for Care and Use of Laboratory Animals. Our facility is accredited and approved by the Association for Accreditation of Laboratory Animal Care International and follows the Public Health Service Policy for the Care and Use of Laboratory Animals (IBC 2023-42; ACUC 21-109). Female BALB/c mice [RRID:MGI:2683685] obtained from the Frederick Cancer Research and Development Center Animal Production Area were used for the *in vivo* studies and housed five per cage. Eight to ten-week-old female WT and $Nos2^{-}$ BALB/c mice were shaved a day prior to tumor injection and then were injected subcutaneously into the fourth mammary fat pad with 200,000 4T1 TNBC cells obtained from ATCC [RRID:CVCL_0125]. The cells were authenticated by the vendor. Upon receipt, the cells were tested for mycoplasma prior to expanding and storage at passage (p.) 8; cells were not used beyond p.30. Tumor measurements began one week after tumor cell injection, using a Vernier caliper and calculated in cubic millimeter volumes according to the following equation.

$$[(\text{short diameter})^2 \times \text{long diameter}]/2$$

Upon reaching tumor size of $100mm^3$, tumor- bearing mice were divided into groups and treatment with 30 mg/L indomethacin (pan-COX inhibitor) in drinking water was initiated and mice were treated for the next seven days. The water was changed every Monday Wednesday Friday for the

duration of the experiment. Mice were monitored daily for signs of gut toxicity; none were observed at this dose. Mice were euthanized per the requirements of the ACUC protocol and committee, and tumor tissues were harvested and flash frozen.

CODEX® Analysis

The CODEX protocol was performed according to Akoya User Manual, revision B.0. Square (22 x 22 mm) glass coverslips (72204-10, Electron Microscopy Sciences) were pre-treated with L-Lysine (#P8920, Sigma, St. Louis, MO [RRID:SCR_008988]) overnight at room temperature. Coverslips were rinsed in distilled water, dried, and stored at room temperature. Fresh frozen tissue blocks were sectioned (10mm) on treated coverslips and stored in a coverslip storage box (Qintay, LLC) at -80°C until further use. CODEX reagents and instrumentation were purchased from Akoya Biosciences (Marlborough, MA). Antibodies labeled for CODEX were CD3 [RRID:AB_3271528], CD4 [RRID:AB_3271537], CD8a [RRID:AB_3271540], CD25 [RRID:AB_312856], CD45 [RRID:AB_3271536], CD274 [RRID:AB_467784], CD279 [RRID:AB_313418], F4-80 [RRID:AB_3271523]. Tissue sections were stained with an antibody cocktail consisting of 0.5-1ml of each antibody per tissue. CODEX assays were performed according to the manufacturer's recommendations. Fluorescent oligonucleotide plates were prepared in black 96-well plates for image acquisition. Each CODEX cycle contains four fluorescent channels (three for antibody visualization and one for nuclear stain). For each cycle, up to three fluorescent oligonucleotides (5 mL each) were added to a final volume of 250 mL of plate buffer (containing Hoechst nuclear stain). For blank (empty) cycles, 5 mL of plate buffer was substituted for fluorescent oligonucleotides. Plates were sealed and kept at 4°C until use. For imaging, the CODEX coverslip was mounted onto a custom-designed plate holder and securely tightened onto the stage of a Keyence BZ-X810 inverted fluorescence microscope. Cycles of hybridization, buffer exchange, image acquisition, and stripping were then performed using an Akoya CODEX instrument. Briefly, that instrument performs hybridization of the fluorescent oligonucleotides in a hybridization buffer, imaging of tissues in CODEX buffer, and stripping of fluorescent oligonucleotides in the stripping buffer. CODEX multicycle automated tumor imaging of was performed using a CFI Plan Apo 20x/0.75 objective (Nikon). The multipoint function of the BZ-X viewer software (BZ-X ver. 1.3.2, Keyence) was manually programmed to align with the center of each tumor and set to 10 Z stacks. Nuclear stain (DAPI, 1:600 final concentration) was imaged in each cycle at an optimized exposure time of roughly 10 ms. The respective channels

were imaged in the automated run using optimized exposure times. Raw TIFF images produced during image acquisition were processed using the CODEX image processer. The processer concatenates Z-stack images, performs drift compensation based on alignment of nuclear stain across images, and removes the out-of-focus light using the Microvolution deconvolution algorithm (Microvolution). The processer also corrects for non-uniform illumination and subtracts the background and artefacts using blank imaging cycles without fluorescent oligonucleotides. The output of this image processing was tiled images corresponding to all fluorescence channels and imaging cycles that were then visualized and analyzed using HALO software (Version 3.3.2541.383, Indica Labs Inc.). Segmentation of cells was performed using the nuclear channel and the cell cytoplasm was defined as a fixed width ring around each nucleus. Nuclear segmentation settings were optimized by visual verification of segmentation performance on random subsets of cells aiming to minimize the number of over segmentations, under segmentations, detected artefacts and missed cells. Cell type Annotation and Differential Marker Analysis Cell populations were gated as follows. Tissues were annotated to exclude edge effect. All nucleated cells were first identified by positive nuclear signals. Definition of immune cell phenotypes based upon biomarker expression as judged by expert visual inspection: $CD8^+$ T_{Eff}, $CD45^+CD3^+CD8^+CD279^-$; murine $CD4^+$ T_{Reg}, $CD45^+CD3^+CD4^+CD25^+$, human, $CD45^+CD3^+CD4^+FOXP3^+$; $PDL1_{Macrophage}$, $CD45^+F480^+CD274^+$.

Cell Culture and Treatment

MDA-MB-231 cells were seeded in 100 mm Petri dishes at a density of 2.62×10^6 cells/dish and incubated overnight at 37 °C in a humidified atmosphere with 5% CO_2. Cells were then serum-starved for 24 h using phenol red-free, serum-free RPMI 1640 (ThermoFisher, Cat# 11835030). Following starvation, cells were treated with 100 U/ml IFN-γ (R&D, cat# 285-IF-100/CF), 10 ng/ml TNF-α (R&D, cat# 10291-TA-020), and 10 ng/ml IL-1β (R&D, cat# 201-LB-010/CF) for 24 or 48 h. Post-treatment, cells were washed twice with cold 1X PBS, scraped, and pelleted by centrifugation at 2000 rpm for 2 min in 1.5 ml microcentrifuge tubes. Pellets were resuspended in 50 µl of M-PER lysis buffer (ThermoFisher, Cat# 78501) supplemented with 1X phosphatase/protease inhibitor cocktail (Cell Signaling, Cat# 5872), shaken at 1200 rpm for 15 min at room temperature, and then incubated on ice for an additional 15 min. Cells were briefly sonicated using the Branson Sonifier 250 (2 sec, constant pulse; output 4; Branson Ultrasonics Corporation, Danbury, CT, USA) and centrifuged at 14,000 rcf for 15 min at 4 °C. Supernatants

were collected and used for downstream analysis. Protein concentration was measured using the BCA assay. Lysates (4 µl) were diluted in 16 µl ddH_2O, matched with equally diluted BSA standards, and assayed in triplicate in a 96-well plate. Plates were incubated for 15 min, and absorbance was read at 562 nm.

Western Blotting

Lysates (50 µg) were mixed with 1X NuPAGE™ LDS Sample Buffer (ThermoFisher, Cat# NP0007) and 50 mM DTT, heated, and loaded onto 10-well 1.0 mm NuPAGE Bis-Tris gels (ThermoFisher, Cat# NP0321BOX) in the XCell SureLock Mini-Cell system (ThermoFisher, Cat# EI0001). Electrophoresis was run at 200 V for 1 h 10 min on ice using MES Running Buffer (ThermoFisher, Cat# NP0002). For protein transfer, PVDF membranes were pre-activated in methanol for 5 min, equilibrated in Transfer Buffer (ThermoFisher, Cat# NP0006), and assembled with the gel in the XCell II™ Blot Module (ThermoFisher, Cat# EI9051). Transfer was performed at 25 V for 1 h 25 min on ice. Membranes were blocked with 5% BSA in TBST for 1 h at room temperature on an orbital shaker (IBI Scientific, USA). Blocked membranes were cut according to target molecular weights and incubated overnight at 4 °C with primary antibodies diluted in 5% BSA: anti-iNOS (Cell Signaling, cat# 20609S, 1:1000), anti-COX2 (Cell Signaling, cat# 12282S, 1:2000), and anti-HPRT (Santa Cruz, cat# sc-376938, 1:1000). After three 10-min washes in 1X TBST, membranes were incubated for 30 min at room temperature with the respective HRP-conjugated secondary antibodies (iNOS: Cell Signaling, cat# 7074S, 1:1000; COX2: Cell Signaling, 1:1000, HPRT, Cell Signaling, cat# 7076S, 1:2000). Following 3 additional 10-min washes, signal was developed using SuperSignal™ West Femto substrate (ThermoFisher, Cat# 34096) for 5 min and imaged using the Amersham Imager 680 (GE Healthcare). Band intensities were quantified using ImageJ.
