# Supplementary figures and images for: Elevated tumor NOS2/COX2 promotes immunosuppressive phenotypes associated with poor survival in ER– breast cancer
Source: JCI Insight. 2025 Jul 15;10(16):e193091. doi: 10.1172/jci.insight.193091 (PMC12406732; doi:10.1172/jci.insight.193091)

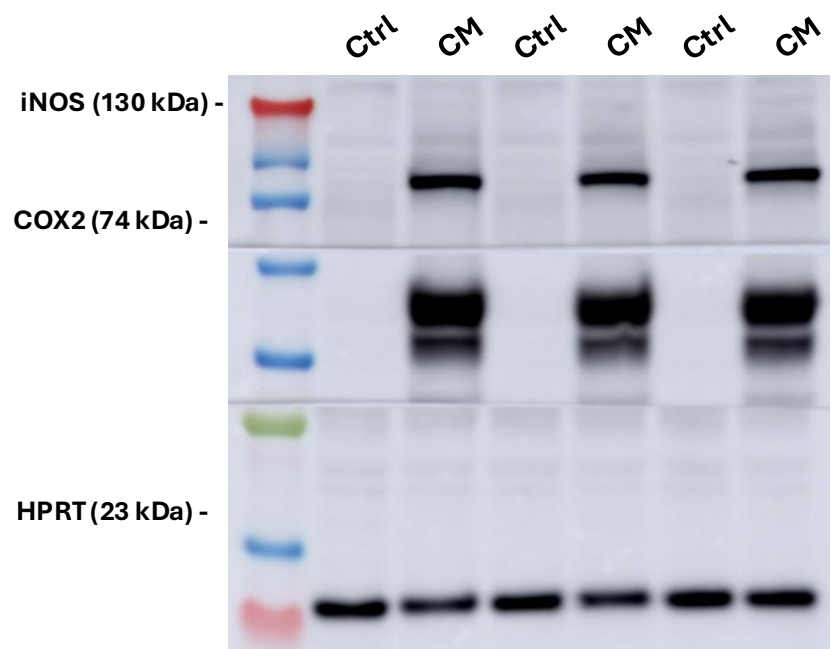

MDA-MB-231 - 24h

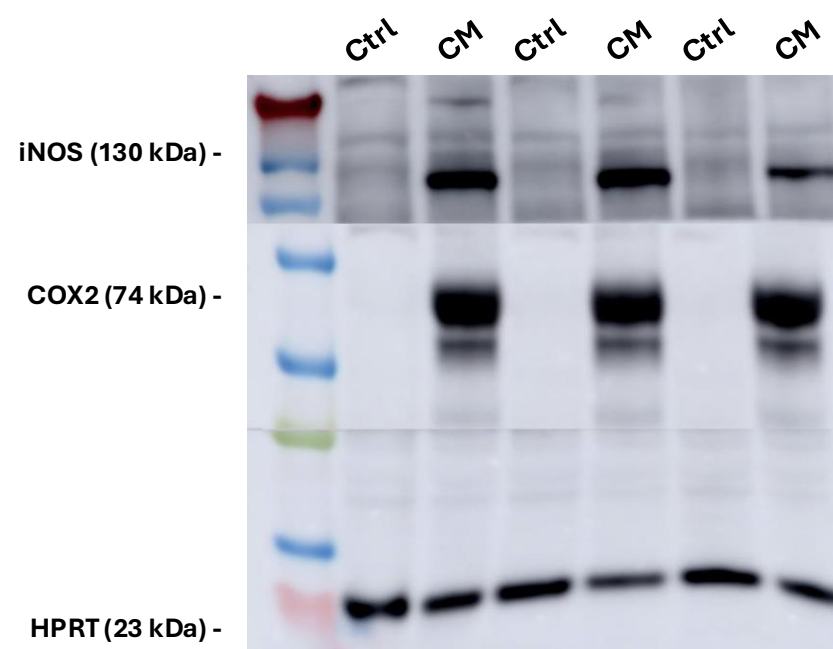

MDA-MB-231 - 48h

Supplement: Unedited blot and gel images [file jciinsight-10-193091-s197.pdf]
